# Low-toxin *Clostridioides difficile* RT027 strains exhibit robust virulence

**DOI:** 10.1080/22221751.2022.2105260

**Published:** 2022-08-08

**Authors:** Farhan Anwar, Bryan Angelo P. Roxas, Kareem W. Shehab, Neil M. Ampel, V. K. Viswanathan, Gayatri Vedantam

**Affiliations:** a School of Animal and Comparative Biomedical Sciences, The University of Arizona, Tucson, AZ, USA; b Department of Pediatrics, The University of Arizona College of Medicine, Tucson, AZ, USA; c Infectious Diseases, Mayo Clinic, Phoenix, AZ, USA; d Department of Immunobiology, The University of Arizona College of Medicine, Tucson, AZ, USA; e BIO5 Institute for Collaborative Research, The University of Arizona, Tucson, AZ, USA; f Research Service, Southern Arizona VA Healthcare System, Tucson, AZ, USA

**Keywords:** *Clostridioides difficile*, toxin, low-toxin, RT027, virulence

## Abstract

*Clostridioides difficile* is a leading cause of healthcare-associated infections worldwide. Currently, there is a lack of consensus for an optimal diagnostic method for *C. difficile* infection (CDI). Multi-step diagnostic algorithms use enzyme immunosorbent analysis (EIA)-based detection of *C. difficile* toxins TcdA/TcdB in stool, premised on the rationale that EIA toxin-negative (Tox^−^) patients have less severe disease and shorter diarrhoea duration. The aim of this study was to characterize toxigenic (i.e. *tcdA/tcdB*-positive) *C. difficile* strains isolated from diarrheic patient stool with an EIA Tox^−^ (i.e. “discrepant”) CDI diagnostic test result. Recovered strains were DNA fingerprinted (ribotyped), subjected to multiple toxin, genome and proteome evaluations, and assessed for virulence. Overall, of 1243 *C. difficile*-positive patient stool specimens from Southern Arizona hospitals, 31% were discrepant. For RT027 (the most prevalent ribotype)-containing specimens, 34% were discrepant; the corresponding RT027 isolates were cytotoxic to cultured fibroblasts, but their total toxin levels were comparable to, or lower than, the historic low-toxin-producing *C. difficile* strain CD630. Nevertheless, these low-toxin RT027 strains (LT-027) exhibited similar lethality to a clade-matched high-toxin RT027 strain in Golden Syrian hamsters, and heightened colonization and persistence in mice. Genomics and proteomics analyses of LT-027 strains identified unique genes and altered protein abundances, respectively, relative to high-toxin RT027 strains. Collectively, our data highlight the robust virulence of LT-027 *C. difficile*, provide a strong argument for reconsidering the clinical significance of a Tox^−^ EIA result, and underscore the potential limitations of current diagnostic protocols.

## Introduction

*Clostridioides difficile* (CD), an anaerobic, spore-forming, Gram-positive bacterium, is the leading cause of healthcare-associated infections in the United States and Europe [1]. In the US, over 223,000 cases of *C. difficile* infection (CDI) occur annually, with associated medical costs estimated at $1 billion [2]. *C. difficile* strains may produce up to three toxins: TcdA (Toxin A), TcdB (Toxin B), and Binary Toxin (CDT) [3]. TcdA and TcdB, encoded within a pathogenicity locus (PaLoc), are glucosyltransferases that target host-cell GTPases including Rho, Rac, and Cdc42 [4]. The resulting inhibition of these small GTPases contributes to cytoskeletal changes, epithelial barrier disruption and, on prolonged exposure, host cell death [3].

The past 20 years have seen the emergence of outbreak-associated strains, particularly those belonging to the ribotype 027 [RT027; also called (REA) type BI or North American Pulsed-Field (NAP) type NAP1]. RT027 strains have been associated with increased prevalence and persistence in clinical settings as well as severe disease [5,6]. The emergence and dominance of this clade have been attributed to various properties such as fluoroquinolone resistance, expression of a variant TcdB toxin [7], and the ability to metabolize the sugar trehalose [8]. While early reports suggested increased toxin production to be a contributing factor to the increased virulence of RT027 strains [9,10], this contention has not been supported by subsequent research in various laboratories [11–13]. RT027 strains are highly variable in the amounts of toxin they produce, and detectable toxin levels are not correlated with disease onset and severity [11,14–16].

There is considerable debate regarding the appropriate diagnostic approach for establishing a CDI [17]. Broadly, diagnostic protocols rely on three assays: (1) nucleic acid amplification tests (NAATs) that detect toxigenic *C. difficile*; (2) enzyme immunoassays (EIA) for glutamate dehydrogenase (GDH), an enzyme expressed in all strains (including non-toxigenic *C. difficile*), and (3) EIA for TcdA and TcdB [18]. A NAAT^+^/TcdAB^+^ or GDH^+^/TcdAB^+^ result is indicative of CDI (Tox+), while CDI is deemed highly unlikely if TcdA/TcdB are not detected (Tox^−^) in the stool. However, CDI cannot be ruled out when the clinical test result is discrepant (NAAT^+^/Tox^−^ or GDH^+^/Tox^−^), and the decision to treat for CDI in these instances is based on clinical judgement [1,19]. Discrepant results may be due to a multitude of factors, including variability in *C. difficile* toxin production [20].

For the current study, our goal was to characterize toxigenic *C. difficile* strains isolated from stool specimens that were discrepant in clinical tests by characterizing their toxin production, and assessing their virulence potential. A large number of discrepant specimens (>31%) in our surveillance included RT027 strains. Detailed assays revealed that clinical EIA test negativity could be explained by low toxin production in these strains. To interrogate pathogenic potential, we, therefore, assessed the virulence of low-toxin RT027 strains using mouse and hamster models of infection, and performed systematic genome and proteome analyses.

## Methods

### Reference strains

We utilized strains BI-1, CD630, VPI 10463, and T7 as reference strains in our study. These were kindly gifted to us by Dr. Dale Gerding (Hines VA Hospital, Hines, IL, USA).

### Specimen acquisition and recovery of *C. difficile*

Stool specimens from patients with clinical suspicion of CDI were obtained over a period of 7 years (2012–2019) from three hospitals in Tucson, Arizona. All specimens were designated “to-be-discarded” and were de-identified prior to acquisition. This study was deemed not to constitute human subjects research and was exempt from full Institutional Review Board approval.

An aliquot of the stool specimen was plated onto taurocholate-cefoxitin-cycloserine-fructose agar (TCCFA) and grown anaerobically for 48 h. Colony PCR-based amplification of *tcdB* was used to confirm presence of toxigenic *C. difficile*. Supplemental Table S1 lists primers and strains used. For the studies described below, and unless otherwise indicated, isolates were grown in 10 mL of Brain–Heart Infusion broth (Bacto BHI; BD, Franklin Lakes, New Jersey, NJ, USA), and cultures maintained in 25% glycerol at – 80°C for long-term storage.

### Spore preparation

*C. difficile* overnight cultures were sub-cultured in 50 mL BHI broth and grown for 14 days. Sub-cultures were then centrifuged at 4000 RPM for 10 min, supernatants decanted, and pellets washed (successive resuspension and then centrifugation) three times with phosphate-buffered saline (PBS). The pellets were then resuspended in sterile water and remaining vegetative bacteria were killed by heat shock at 65°C for 15 min. Spores were purified via at least three additional washes in distilled water, or via Ficoll gradient centrifugation as previously described [21], and stored in water at 4°C.

### Ribotyping and colony PCR

Overnight BHI broth cultures of *C. difficile* strains were centrifuged and resuspended in 1 mL of Tris-EDTA (pH 7) and processed for genomic DNA extraction and ribotyping PCR as per published protocols (Supplemental Table S1) [22,23]. PCR products were separated via capillary electrophoresis, and the ribotype established via comparison to the online database Webribo [24].

The presence of toxin genes was verified via PCR amplification of sequences specific to *tcdA*, *tcdB,* and binary toxin genes (Supplementary Table S1), respectively, as previously described [23,25]. Isolated *C. difficile* colonies on TCCFA were transferred to 25 µL of PCR-Lyse™ (Epicentre, Madison, WI, USA) solution, incubated at 99°C for 5 min, and immediately placed on ice. The sample was centrifuged, and the supernatant was used for PCR analysis.

### Toxin analysis

Clinical and reference *C. difficile* strains were grown in 50 ml BHI broth for 72 h. The cultures were then centrifuged, and cell-free supernatants were clarified using 0.22 µm filters (Argos Technologies, Elgin, IL, USA). Toxin levels in 50 μL of the supernatants were assessed using the clinically-common Techlab^®^ *C. difficile* Tox A/B II™ EIA kit (Techlab, Blacksburg, VA, USA) as per manufacturer’s instructions, and optical density measured at 450 nm. Total protein concentration was assessed using the Pierce™ BCA Protein Assay Kit (ThermoFisher Scientific, Waltham, MA, USA), and toxin levels are reported as OD_450_/mg total protein. All data were collected in biological triplicate.

Immunoblotting was also performed on select isolates. Following overnight growth and sub-culture in 50 mL BHI broth for 72 h, the cultures were centrifuged, and 30 mL of the supernatant was concentrated using a 100 kDa filter (Amicon Ultra, Millipore, Bedford, MA, USA). 50 μg of protein from the concentrated supernatants were used toxin immunoblotting. Briefly, the protein samples were separated by SDS-PAGE on 7.5% TGX-PAGE gels (BioRad, Hercules, CA, USA) at 100 V for 90 min. Proteins were transferred onto nitrocellulose membranes using the Trans-Blot Turbo Transfer System (BioRad, Hercules, CA, USA) and immunoblotted with rabbit monoclonal IgG against TcdA and TcdB (AbCam, Waltham, MA, USA) at 1:8000 concentration overnight at 4°C. Secondary goat anti-rabbit HRP-conjugated IgG was used at 1:10,000 concentration for one hour at room temperature and bands were visualized using the SuperSignal™ West Femto Substrate (Thermo Fisher, Waltham, MA, USA) on a ChemiDoc Imaging System (BioRad, Hercules, CA, USA).

### Cytotoxicity assays

Cultured Vero fibroblasts were propagated in 96-well plates with Eagle’s Minimum Essential Media (EMEM; Corning Life Sciences, Corning, NY, USA) containing 10% fetal bovine serum (FBS; Corning Life Sciences, Corning, NY, USA). Clarified *C. difficile* supernatants (described above) sequentially diluted in EMEM + FBS (varying amounts of total toxin) were added to each well and incubated for 3 h, unless otherwise specified, at 37°C with 5% CO_2_. In additional assessments, culture supernatants with identical total toxin amounts were also added to Vero cells and observed after 1, 2, 6, and 18 h post-treatment. Cells were visualized using a bright field microscope (Olympus BHTU; Olympus, Center Valley, PA, USA), and the degree of cell rounding scored on a scale of 1 (< 25% rounded cells) to 4 (>75% rounded cells). TcdA/TcdB-dependent rounding was verified via neutralization with anti-TcdA and anti-TcdB monoclonal antibodies (Abcam, Waltham, MA, USA). A minimum of 10 fields per treatment, with three independent replicates, were assessed.

### Antibiotic susceptibility assays

Susceptibility of select *C. difficile* isolates to cefotaxime, rifampicin, levofloxacin, metronidazole and vancomycin was determined using Clinical and Laboratory Standards Institute (CLSI) protocols (M11-A8) [26]. Pure cultures were grown overnight, sub-cultured to 0.5 McFarland standard, and plated on Brucella agar with hemin, vitamin K1, and 5% horse blood (BD, Franklin Lakes, New Jersey, NJ, USA). Antibiotic susceptibility was determined using the bioMérieux Etest^®^ platform (bioMérieux, Durham, NC, USA).

### Golden Syrian hamster infections

Golden Syrian hamsters were used to assess virulence of select *C. difficile* strains [27,28]. Seven to eight-week-old male hamsters (90–100 g; Charles River, Wilmington, MA, USA) were orally administered clindamycin (30 mg/kg) (Pfizer, New York, NY, USA) 72 h prior to infection. For pilot studies, animals (2 hamsters/strain) were orally infected with 100 spores of *C. difficile* LT-027 strain (GV106, GV135, or GV148) or a high-toxin RT027 strain (BI-1) [29]. For fully-powered studies, animals were infected with 100 spores of the LT-027 strain GV148 (*N* = 8), BI-1 (*N* = 6), or a non-toxigenic strain (T7; *N* = 2). In all studies, animals were monitored for overt disease every 6–12 h (diarrhoea, inappetence, ruffled fur, lethargy). Surviving animals were humanely euthanized at the end of the study using a commercial euthanasia solution (270 mg/kg sodium pentobarbital: Merck, Kenilworth, NJ, USA). *C. difficile* burden in stool and cecal contents was determined by plating on TCCFA. Cecal toxin burden was measured by centrifuging the cecal content at 14,000 RPM for 20 min, and using the surface 500 µL for toxin measurements as described above.

### Mouse infections

Intestinal colonization of select *C. difficile* strains was assessed using a non-lethal mouse model of infection [30]. 10-week-old male C57BL/6 mice (Jackson Laboratories, Bar Harbor, ME, USA) were pre-treated with cefoperazone (0.5 mg/mL) in the drinking water for 10 days, followed by intraperitoneal clindamycin (10 mg/kg) (Pfizer, New York, NY, USA) on day −1. The animals were administered 10^6^ spores of BI-1, GV106, GV135, or GV148, via oral gavage on day 0 (*N* = 5/group). Animals were weighed once daily and monitored twice daily for signs of stress. Surviving animals were humanely euthanized at the end of the study using a commercial euthanasia solution described above. Stool pellets or cecal content were collected daily, resuspended, homogenized in PBS, serially diluted, and plated onto TCCFA for isolation and bacterial burden (CFU/g of stool or cecal tissue).

### Serum collection and immunoblotting of surface proteins

Mice infected with high toxin strains (BI-1, GV599) or LT-027 strains (GV148, GV1002) were humanely euthanized on day 14 post-infection, and blood was collected via cardiac puncture. Serum was isolated by centrifuging the blood for 15 min at 400 rpm. HALT protease (ThermoFisher Scientific, Waltham, MA, USA) was added to the serum and stored at −80°C.

*C. difficile* surface proteins were extracted for immunoblotting as previously reported [21]. Briefly, each strain was grown overnight in 50 mL of BHI media. The cultures were centrifuged, and the pellets were resuspended in 500 µL of 0.2 M glycine (pH2.2). 30 µg of surface extract was separated on a 4–20% gradient acrylamide gel and transferred to a PVDF membrane. These were probed with collected serum at a 1:500 dilution overnight, and an HRP-conjugated goat anti-mouse secondary antibody (1:10,000 dilution; Abcam, Waltham, MA, USA) for 1 h; the blots were developed using SuperSignal West Femto chemiluminescent substrate (ThermoFisher Scientific, Waltham, MA, USA) and imaged with a Chemi-doc Imager (Bio-Rad, Hercules, CA, USA).

### Comparative genomics and proteomics

Whole genome sequencing of 15 LT-027 and 3 RT027 isolates was performed using the Illumina MiSeq platform at the University of Arizona Genomics Core. The sequences were assembled using the CLC Genomics Workbench (Qiagen, Hilden, Germany) and annotated using RAST (NMDPR, Chicago, Illinois) (Supplemental Table S2). Alignment-free composition vector tree analysis of these genomes, and 10 publicly-available RT027 genomes, was performed using CVTree 3.0 [31] and visualized using the Interactive Tree of Life v4 (ITOL; https://itol.embl.de/) [32]. Eleven LT-027 strains were compared to previously published and publicly available RT027 genomes (*N* = 10). For additional genomics rigour, a second clinically-relevant *C. difficile* ribotype (RT106) wherein isolates are low- or high-toxin-producing, was similarly assessed; therefore five LT-106 strains and four high-toxin RT106 strains were sequenced, and composite vector trees created. KEGG analysis was done on unique genes found in the low-toxin RT027 strains. Briefly, amino acid sequences were uploaded into the BlastKOALA query tool (https://www.kegg.jp/blastkoala/) to identify any genes identical to known KEGG-annotated genes based on homology. Our sequences were compared to the four *C. difficile* KEGG-annotated genomes of CD196, R20291, 630, and 630Derm (“Δ*erm”*).

For comparative proteomics analyses, strains were grown in BHI media overnight, and then sub-cultured 1:50 in BHI. Samples were collected at mid-logarithmic growth phase and processed using the iTRAQ (isobaric tagging for relative and absolute quantitation) analysis as previously reported [33,34]. The *C. difficile* strain BI-1 protein database (Refseq assembly GCF_000211235.1) was used as the reference. Differentially-abundant proteins were identified via multiple stringent statistical tests as described below.

### Statistical analyses

XLSTAT was used for statistical analysis and generating PCA plots. For bacterial burden, Student’s *t*-tests and ANOVA was used to determine differences between strains. For studies with large number of observations for various time points (such as cytotoxicity analyses), goodness-of-fit statistical analyses were performed using ANOVA followed by stringent Tukey tests. *p* values of <.05 were considered significant.

For proteomics, additional tests established significance of protein abundance changes. Specifically, three parameters had to be satisfied prior to a protein being categorized as differentially abundant [33]: (1) a (stringent) false discovery rate (FDR) of 1%, (2) a fold change cutoff determined by using a technical replicate in each experiment; and (3) hypergeometric testing, similar to the Fisher’s exact one-tailed test, where, in addition to the parameters described above, a significance of *p* < .05 had to be achieved for differentially abundant proteins to be classified as such.

For all animal studies, *χ*^2^ analyses determined the percentage of animals colonized with *C. difficile*, the time interval between *C. difficile* challenge and colonization, and time between *C. difficile* challenge and death. Additionally, Kaplan–Meier survival curves were generated, followed by Log-Rank tests for *post hoc* analyses. Significance was determined by ANOVA, where the goodness of fit and standardized coefficients of variance were derived.

### Ethics statement

All *in vivo* studies were carried out in strict accordance with the recommendations in the Guide for the Care and Use of Laboratory Animals of the National Institutes of Health. All animal studies were approved by the Institutional Animal Care and Use Committee of the University of Arizona (Protocol Number GV 14-526; PHS Assurance Number A3248-01; USDA Registration Number 86-R-0003). For human stool specimens, all samples were designated “to-be-discarded” and were de-identified before acquisition; this study was performed under an Institutional Review Board (IRB) Non-Human Subjects Research Data Use Committee Approval NRDUC # 1707612129.

## Results

### *C. difficile* isolates from discrepant specimens express low levels of glucosylating toxins

From 2012 to 2019, we isolated, purified and ribotyped 1243 *C. difficile* isolates from patient stool specimens obtained from three different Tucson-area hospitals. Over this 7-year time period, RT027 was the most prevalent ribotype [19.2% (*n* = 239)]. Restricting analysis to those specimens for which clinical EIA results were available (*n* = 999), 39.04% (*n* = 390) were deemed to be “discrepant” (NAAT^+^/Tox^−^). Of the EIA-tested specimens specifically harbouring RT027 strains, 34% (*n* = 75) were discrepant.

**Figure 1.**
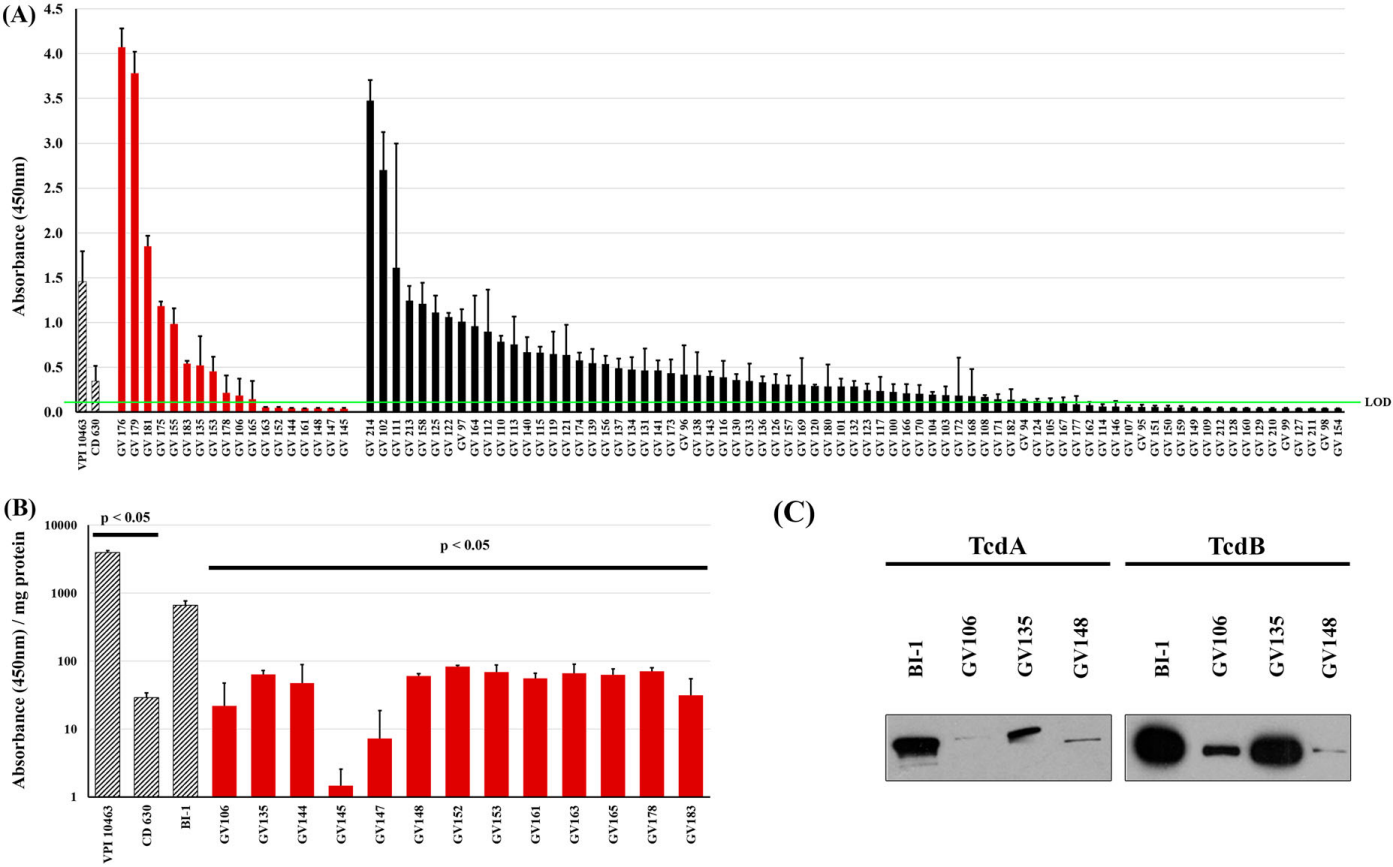
Clinical *C. difficile* strains display high variability in toxin production. (A) Clarified supernatants from 93 *C. difficile* strains were measured for toxin with a TcdA/B EIA kit, normalized to volume. There is high variability in toxin abundance, even among RT027 strains (red bars; black bars include all other ribotypes). The high-toxin producer VPI 10463 (at 1:100 dilution) and low-toxin producer CD630 are included for comparison (hashed bars). Thirty strains registered readings below the limit of detection (LOD, green line = OD_450_ < 0.12). (B) Toxin per mg of supernatant from 13 RT027 strains previously shown in (A) had nearly 10-fold lower TcdA/B amounts relative to the comparator RT027 strain BI-1. Additionally, BI-1 produced significantly more toxin than CD630 and significantly less than VPI 10463. (*p* < .05; two sample, two-tailed *t*-test). (C) Immunoblots of supernatants (30 μg/lane) from these strains confirmed lower abundance of TcdA and TcdB in supernatants of three representative LT-027 strains relative to BI-1.

Ninety-three unique *C. difficile* isolates from corresponding sequentially-obtained unique specimens were first assessed for their ability to produce TcdA and TcdB following in vitro culture. Cell-free supernatants from stationary growth-phase cultures (72 h) were assayed for TcdA/TcdB abundance via a clinically-employed EIA (Figure 1A). The low-toxin producing strain CD630 and high-toxin-producing strain VPI 10463 were used as comparators [9,35]. The 93 *C. difficile* strains produced highly divergent amounts of TcdA/TcdB. Toxin levels for 74/93 isolates (79.5%) were at, or lower than, CD630, with 30 (32.3%) being below the limit of detection (OD_450_ < 0.12). Notably, and consistent with other reports, there was substantial variability in toxin amounts produced by the RT027 strains (*n* = 18) in this sample set [20]. Specifically, 13/18 (72%) RT027 strains produced total toxins that were at, or below, that of CD630 (Figure 1A). Even after normalization for protein amounts, the 13 RT027 strains produced nearly 10-fold lower toxin levels relative to the reference, RT027 comparator BI-1 (Figure 1B). Immunoblot analysis of select RT027 isolates confirmed these decreased TcdA/TcdB levels compared to BI-1 (Figure 1C). We designated these RT027 strains that consistently produced >10-fold less toxin than BI-1 as “low-toxin 027” or “LT-027” strains. Importantly, LT-027 strains had similar growth kinetics as BI-1 (not shown). Taken together, our findings reveal that, irrespective of ribotype, toxigenic *C. difficile* strains are highly divergent in the amounts of toxin they secrete. Thus, the inherently low levels of toxins produced by some *C. difficile* strains likely contribute to the NAAT^+^/Tox^−^ result in clinical diagnostic assays.

### Systematic assessment of toxin production by low-toxin 027 *C. difficile* isolates

**Figure 2.**
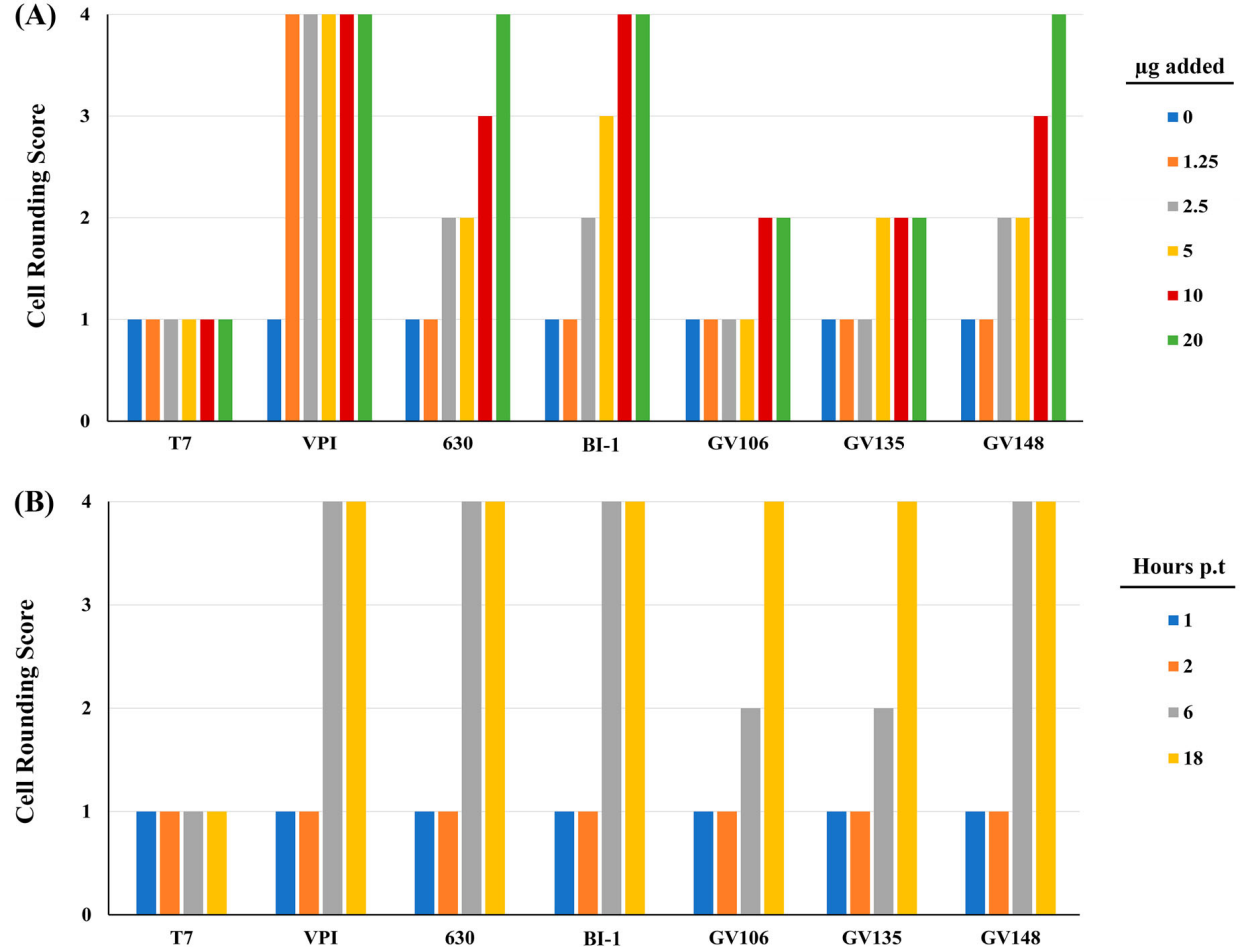
Cytotoxic activity of LT-027 strains is comparable to that of BI-1. (A) Vero fibroblasts were exposed to culture supernatants containing varying TcdA/B concentrations (0, 1.25, 2.5, 5, 10, and 20 µg) for 3 h; cell rounding was scored 1 (< 25% rounded cells) to 4 (>75% rounded cells). BI-1 and VPI 10463 (high-toxin producing strains) exhibited cell rounding at lower concentrations than supernatants of LT-027 strains GV106, GV135 and GV148. (B) Supernatant volumes were normalized to total toxin amounts, and frequency of rounded cells were counted at specified time post-inoculation (1, 2, 6, and 18 h). LT-027 strains displayed comparable, or slightly slower, cytotoxicity kinetics as VPI 10463, BI-1, and CD630. Cell counts were averaged from 10 fields per condition; triplicates of these sets resulted in the same average score and, therefore, no standard error is reported.

Of these 13 LT-027 strains above, we interrogated three strains (GV106, GV135, and GV148), collected from different hospitals, in detail using *in vitro* and *in vivo* assessments. First, we considered the possibility that LT-027 strains, albeit low-toxin producers, could be secreting more potent toxins, and we, therefore, used cytotoxicity assays to assess toxin activity. Consistent with its higher toxin levels, BI-1 supernatants induced Vero cell rounding at lower protein concentrations than the LT-027 strains GV106, GV135, GV148 (Figure 2A). When normalized to total toxin amounts, however, LT-027 strains and BI-1 induced Vero cell rounding at similar rates (Figure 2B). Collectively, these data suggest that while LT-027 strains produce substantially less TcdA/TcdB than BI-1, the toxins are similar in their cytotoxic activity to host cells. This is consistent with our genomics assessments showing that the PaLoc region, which includes *tcdA* and *tcdB*, is 100% conserved between BI-1 and the LT-027 strains (discussed below).

We next considered the possibility that distinct antibiotic resistance profiles might confer a selective survival advantage to LT027 strains enabling higher bacterial burdens *in vivo* (effectively increasing toxin amounts). Antibiotic susceptibility assessments, however, revealed uniform susceptibility of all RT027 strains tested (LT- or otherwise) to vancomycin, the current “gold standard” agent for CDI treatment (Supplemental Table S3). While LT-027 strains exhibited variable susceptibility to other antibiotics, they did not collectively display a specific resistance pattern to distinguish them from BI-1.

### LT-027 *C. difficile* isolates cause lethal disease in hamsters

**Figure 3.**
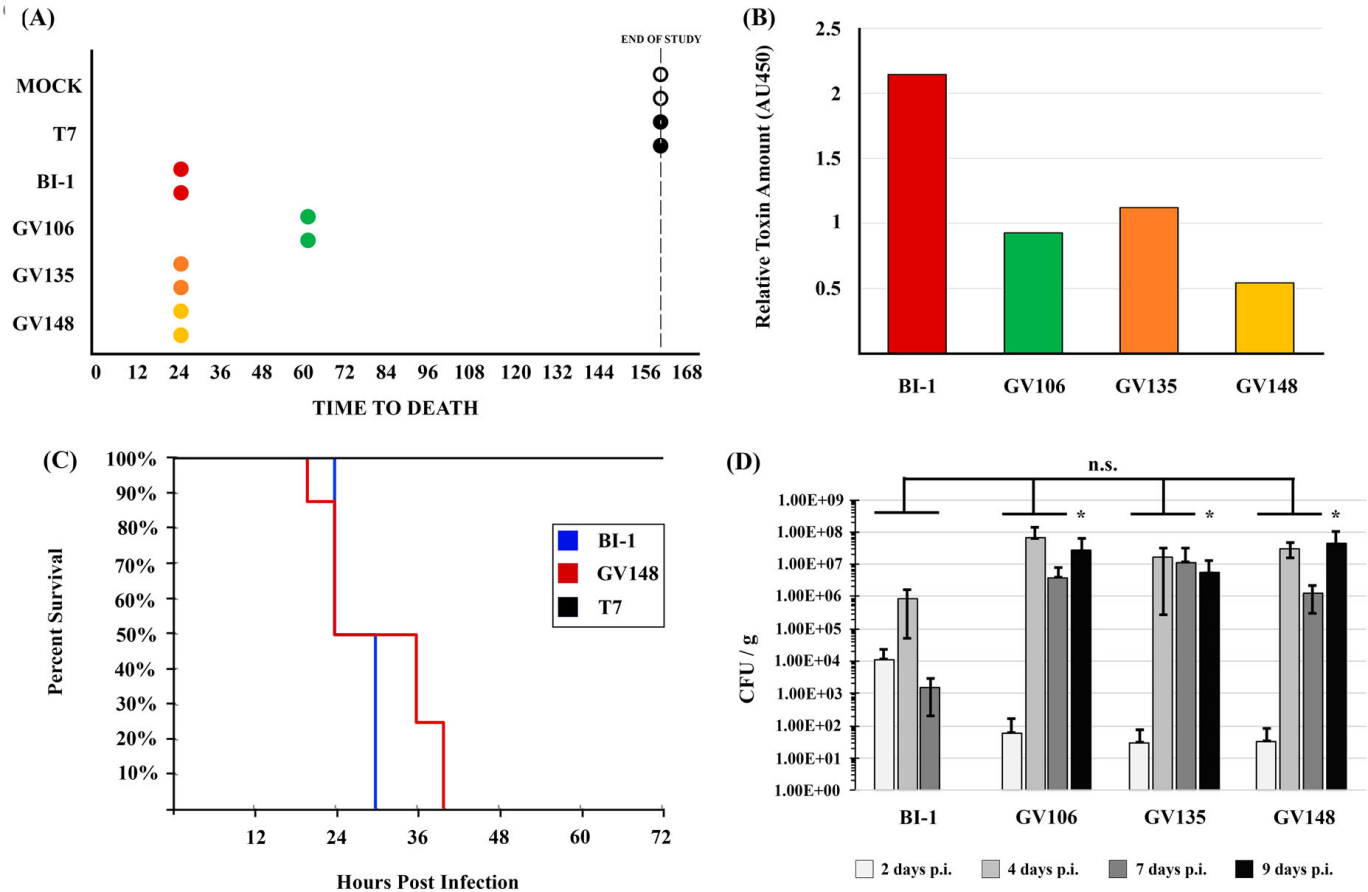
LT-027 strains are virulent and exhibit a colonization advantage *in vivo*. (A) Pilot study of Golden Syrian hamsters (*n* = 2 / group) infected with BI-1 (red), T7 (non-toxigenic strain; black), GV106 (green), GV135 (orange), or GV148 (yellow). All LT-027 strains succumbed to disease with a timeframe comparable to that of BI-1-infected animals. Animals infected with the non-toxigenic T7 strain did not display any symptoms and survived the infection. (B) Average cecal toxin measurements from all infected hamsters with BI-1 (red), T7 (non-toxigenic strain; black), GV106 (green), GV135 (orange), or GV148 (yellow). Clinically-employed TcdA/TcdB EIA kit used. Cecal toxin concentrations were lower in the LT-027-infected animals relative to BI-1, normalized to volume. (C) Golden Syrian Hamsters infected with BI-1 (blue, *n* = 6), GV148 (red, *n* = 8), or T7 (black, *n* = 2). No statistically significant difference in time-to-death between GV148 and BI-1 (log-rank, *p*-value < .05). (D) C57BL/6 mice infected with BI-1 or select LT-027 strains (*n* = 5 / group); burden was measured daily. LT-027 colonized mice robustly and persisted longer compared to BI-1. Statistically-significant burden difference on Day 9 between LT-027 strains and BI-1 (*p* < .05 independent *t*-test, ANOVA; asterisk).

The correlation between stool toxin levels and symptomatic CDI continues to be actively debated [11,36,37]. This prompted us to assess if strains isolated from discrepant clinical specimens, and that consistently produce low levels of toxin, can cause disease in the Golden Syrian hamster model of acute CDI. In a pilot study, hamsters were infected with 100 spores of LT-027 isolates (GV106, GV135, or GV148), BI-1, or the non-toxigenic strain, T7. The LT-027 strains GV135 and GV148, like the reference BI-1 strain, caused 100% lethality within 24 h of infection; LT-027 strain GV106 also induced 100% lethality, but with slightly delayed kinetics (60 h post-infection) (Figure 3A). All toxigenic strains induced hallmark CDI clinical signs including diarrhoea, rapid weight loss, lethargy, inappetence, and alterations in gross intestinal pathology. Cecal toxin abundance of TcdA/TcdB was, however, lower in LT-027 infected animals compared to BI-1-infected animals (Figure 3B). Finally, all tested LT-027 strains continued to display the low toxin phenotype following recovery and propagation from infected hamster stool (data not shown).

Next, we performed a powered animal study comparing the virulence of the LT-027 isolate GV148 (*N* = 8) to BI-1 (*N* = 6). All GV148-infected animals displayed similar disease progression and succumbed to disease between 24 and 40 h post-infection (average time to death was 32 h; Figure 3C). BI-1-infected animals showed a trend towards earlier death (24–30 h), but this was not statistically significant relative to the mortality kinetics for GV148-infected animals. As expected, control hamsters infected with the non-toxigenic strain T7, survived the infection. Collectively, our hamster assessments revealed that, despite producing a >10-fold lower level of toxins, LT-027 strains exhibited robust virulence in an acute CDI model, with disease progression indistinguishable from that of high-toxin (BI-1) strain infection.

### LT-027 *C. difficile* are shed in greater numbers and persist longer than strain BI-1 in infected mice

In the hamster model, *C. difficile-*infected animals succumb to infection within 48 h, potentially masking strain-specific differences in disease manifestation. We, therefore, used a non-lethal mouse model to assess colonization differences between LT-027 strains and BI-1. Antibiotic-treated C57BL/6 mice were orally administered either PBS, or spores of strains BI-1, or an LT-027 strain (GV106, GV135, or GV148). *C. difficile* was detected in the stools of all animals by day 2 post-infection, with a 100-fold higher initial burden in mice infected with BI-1 compared to those treated with LT-027 (Figure 3D). Although stool *C. difficile* burdens increased in all animals by day 4 post-infection, we observed higher pathogen counts for LT-027-infected mice, starting day 4, relative to BI-1. Subsequently, BI-1 burdens in the stool decreased by day 7, with complete clearance by day 9 post-infection. In sharp contrast, LT-027 strains persisted at high levels in the stool on days 7 and 9 post-infection. These results suggest that LT-027 strains colonize more robustly, and persist longer, than BI-1.

### *C. difficile* LT-027 strains express unique proteomes

The increased stool burdens for GV106, GV135, or GV148, relative to BI-1 suggested that LT-027 strains may have unique characteristics, beyond decreased toxin levels, that facilitate colonization. To identify contributing factors, we performed comparative, quantitative, mass spectrometry-based proteomics analyses on three LT-027 isolates (GV106, GV135, and GV148) relative to BI-1. Despite belonging to the same clade (lineage) as BI-1 (RT027), there were marked protein abundance differences between each of the LT-027 isolates and BI-1 at mid-logarithmic growth phase (Supplemental Figure S3, Supplemental Table S6–S8). In GV106, one protein, a Sigma 54-interacting transcription anti-terminator, was significantly upregulated, and 63 proteins were downregulated (Table S6). GV135 had five proteins significantly upregulated, and five proteins downregulated (Table S5). GV148 had eight proteins significantly upregulated, and six proteins downregulated (Table S4).

Importantly, and collectively, there were notable alterations in the abundances of cell wall-associated proteins in the LT-027 strains. GV135 and GV148 had significantly increased abundance of D-alanyl-D-serine ligase (CDBI-1_08005) and D-alanyl-D-alanine carboxypeptidase (CDBI-1_08010) (Supplementary Table 4 and 5). In GV106 D-alanyl-D-serine ligase and D-alanyl-D-alanine carboxypeptidase were downregulated compared to BI-1, though not significantly so (Supplementary Table 6). Additionally, all three strains had decreased abundance of D-alanyl-D-alanine ligase (CDBI-1_06520) and, in the case of GV106, SlpA was downregulated. Further, the S-layer precursor protease, Cwp84 (CDBI-1_13585), was less abundant in all tested LT-027 strains than in BI-1.

### *C. difficile* LT-027 strain express unique surface antigens

**Figure 4.**
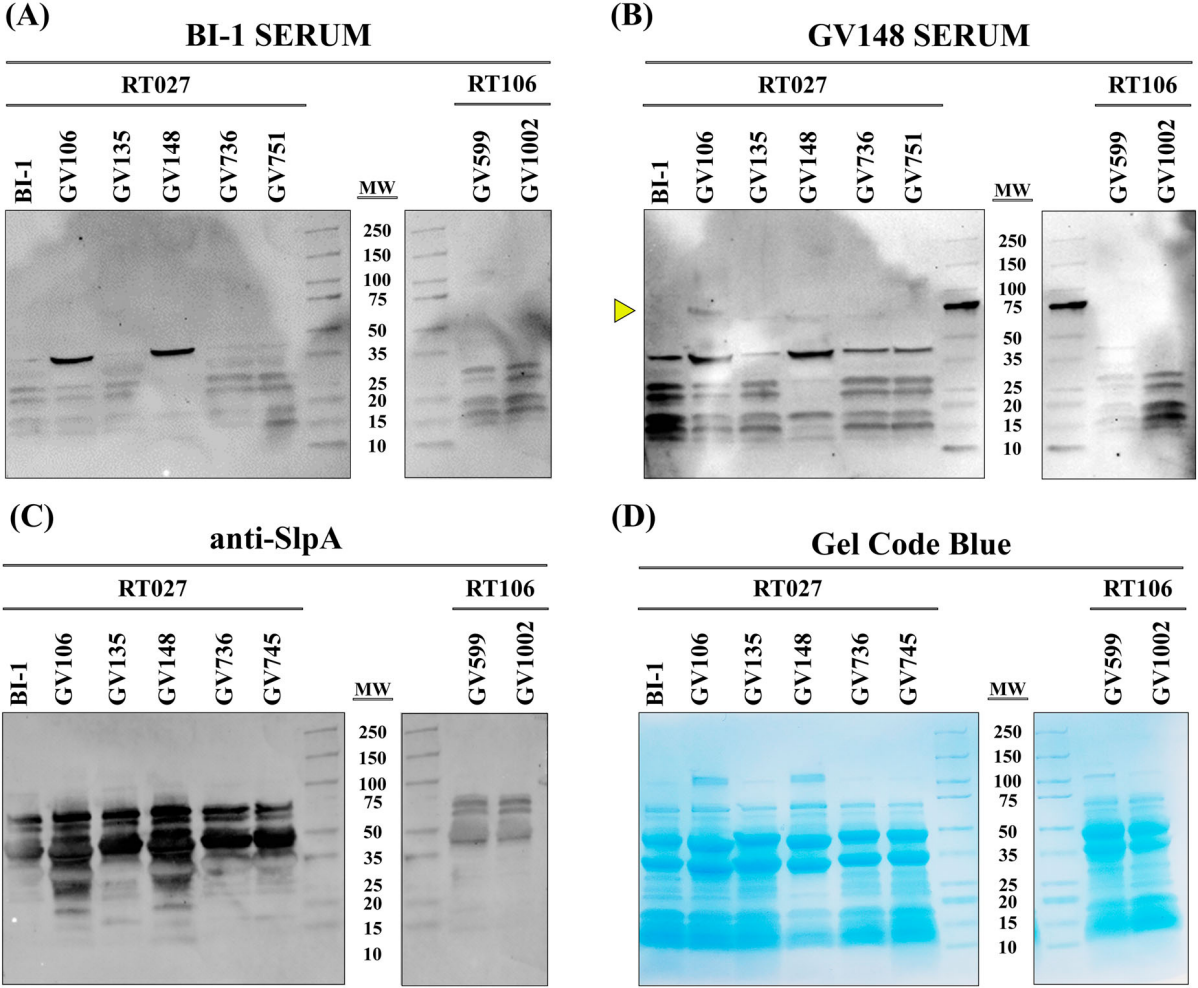
LT RT027 strains exhibit a unique antigen profile. *C. difficile* surface layer extracts (30 µg/lane) from high-toxin RT027 (BI-1 and GV751) and LT-027 strains (GV106, GV135, GV148, and GV736), as well as LT-106 (GV1002) and high-toxin RT106 (GV599), probed with serum isolated from mice infected with the high-toxin RT027 strain BI-1 (A), LT-027 strain GV148 (B), or anti-SlpA antiserum (C). Protein staining (Gel Code Blue) to verify overall Surface-Layer Protein (SLP) banding and quantitation (D). GV148 antiserum detects a unique band (yellow arrow, ∼75 kDa) in surface extracts of LT-027, but not the high-toxin strain BI-1. Further, this band is not detected when probed with BI-1-infected mouse serum. GV599 and GV1002 are high- and low-toxin RT106 strains, respectively, and used as controls (non-RT027 clade).

The marked alteration in abundance of select LT-027 proteins, coupled with increased mouse gut colonization, suggested possible differences in bacterial cell surface architecture and, consequently, antigen profile, relative to BI-1. To assess this, membrane-bound protein fractions of LT-027 and comparator strains were extracted, and equivalent protein amounts (Figure 4D) were probed with serum harvested from infected mice. Serum from BI-1-infected mice was able to recognize *C. difficile* surface antigens from membrane-bound fractions of LT-027 strains (Figure 4A). However, serum from LT-027-infected animals recognized a unique cell-surface protein (∼75 kDa) only in LT-027 membrane fractions, but not in the high-toxin RT027 (BI-1) membrane fraction (Figure 4B; yellow arrow). This moiety is specific to the RT027 clade, since it was not detected when probing membrane fractions of high-toxin (GV599) and low-toxin (GV1002) isolates from an unrelated *C. difficile* lineage (RT106). Similarly, low-toxin RT106-infected mouse serum recognized unique proteins only in low-toxin RT106 strains, and not in a lineage-matched high-toxin RT106 strain, or in any RT027 strain (Supplemental Figure S6A and S6B).

Since SlpA is the most abundant surface protein in *C. difficile* and is involved in host-cell adhesion, we additionally probed *C. difficile* membrane fractions with anti-SlpA antisera (Figure 4C). High molecular weight and low molecular weight SlpA was observed at similar levels in all RT027 strains irrespective of toxigenic potential. Additionally, the corresponding total surface layer protein (SLP) banding patterns were similar between all strains in a clade, but varied between clades (RT027 versus RT106; Figure 4D), indicating ribotype-specificity of cell-surface architecture.

### LT-027 *C. difficile* share unique genomic content not found in the reference RT027

To determine if there is genetic content unique to the low-toxin strains, we compared whole genomes of 17 LT-027 strains to the reference RT027 strain BI-1; all isolates had similar genome sizes (4.1–4.28 MB). LT-027 strains harbuored no obvious unique DNA islands, or toxin/antibiotic resistance genes. All LT-027 strains harboured binary toxin. In all sequenced LT-027 strains, the PaLoc was 100% conserved relative to BI-1, including an 18 bp deletion in *tcdC*. Known regulators of toxin gene expression (TcdR, CodY, CcpA, SigD, and RstA [38]) were also 100% identical among all RT027 strains.

We constructed an alignment-free composite vector tree (CVTree 3.0) from 15 sequenced LT-027 strains, 3 GV-RT027 strains, and 10 publicly available RT027 strains. We observed that LT-027 strains (red) cluster separately from reference/high-toxin strains (black) (Supplemental Figure S2). This suggests there are common genetic elements shared between LT-027 strains. To elucidate this, we generated a pan-genome for LT-027 strains (*N* = 10) and compared this to the pan-genome of high-toxin RT027 strains (*N* = 10), including geographically-distinct, outbreak-associated strains R20291, CD196, BI-1, and BI-8. This analysis revealed 66 genes unique to the low-toxin pan-genome (Supplemental Table S4). Of these, 10 genes were predicted to encode cell surface-associated molecules (Table 1). It is possible that one, or several, of these genes are responsible for the increased colonization seen in the murine model. KEGG analysis of these 66 genes indicated that 44 (67%) were functionally categorized by amino acid sequence comparison to KEGG-annotated *C. difficile* genomes (Supplemental Table S6). These genes were not concentrated in one functional group, but rather dispersed among 15 functional subcategories. However, two genes were identified to be involved in peptidoglycan biosynthesis and degradation; these are fig|1496.1250.peg.1256 (*N*-acetylmuramoyl-l-alanine amidase) and fig|1496.1250.peg.2338 [*murA* (UDP-*N*-acetylglucosamine 1-carboxyvinyltransferase)] (Supplemental Table S4 and S5).

**Table 1.** Cell-wall-associated genes unique to LT-027 strains

Gene ID	RAST function
fig|1496.1250.peg.845	d-aminopeptidase dipeptide-binding protein DppA (EC 3.4.11.-)
fig|1496.1250.peg.1256	*N*-acetylmuramoyl-l-alanine amidase (EC 3.5.1.28)
fig|1496.1250.peg.1416	Putative membrane protein (putative phage infection protein)
fig|1496.1250.peg.2205	Cell wall-binding protein
fig|1496.1250.peg.2413	Peptidoglycan *N*-acetylglucosamine deacetylase (EC 3.5.1.-)
fig|1496.1250.peg.2338	UDP-*N*-acetylglucosamine 1-carboxyvinyltransferase (EC 2.5.1.7)
fig|1496.1250.peg.2560	ABC transporter, permease protein
fig|1496.1250.peg.2670	Arsenic efflux pump protein
fig|1496.1250.peg.3097	Membrane component of multidrug resistance system
fig|1496.1250.peg.3269	Foldase protein PrsA precursor (EC 5.2.1.8)

Note: These are genes found in only LT-027 strains and not in high toxin-producing RT027 strains.

The studies above reveal distinct differences between low- and high-toxin strains within a single *C. difficile* lineage (RT027), raising the possibility that the observed phenotypes and genotypes are ribotype-specific. Therefore, we assessed high- and low-toxin-producing strains of another USA-dominant *C. difficile* lineage, RT106. Genetic differences between low-toxin RT106 (LT-106) and high-toxin RT106 strains were not as stark as their RT027 counterparts, with less discrete clustering (Supplemental Table S4), and fewer unique genes (*n* = 18) identified in the LT-106 pangenome compared to the high-toxin RT106 pangenome (Supplemental Table S9). Only one gene (predicted to encode an RNA-binding protein) was common between the LT-027 and LT-106 pangenomes. Like the LT-027 strains, however, the LT-106 strains do harbour a cell-wall associated gene not found in high-toxin RT106 strains. Specifically, LT-106 strains harbour a peptidoglycan transglycosylase gene (fig|1496.6344.peg.2047) (Supplemental Table S9). Similar to the LT-027 strains, however, LT-106 strains also exhibited increased persistence in mice (Supplemental Figure S5). Furthermore, immunoblots using serum of LT-106 infected animals revealed unique bands in LT-106 membrane fractions but not in high-toxin RT106, or any RT027, membrane fractions (Supplemental Figure S6).

## Discussion

While it is well established that the glucosylating toxins TcdA and TcdB are essential for *C. difficile* virulence [20,27,39–42], there is no evidence to suggest that a threshold toxin amount is required to elicit diarrheagenic CDI symptoms. Our studies confirm that clinical isolates, including RT027 strains, vary significantly in the production of TcdA and TcdB. Toxigenic *C. difficile* strains were recovered from >30% of toxin EIA-negative stool specimens, all from symptomatic patients. Similarly, Erb et al noted that 42.9% (*N* = 206) of patient samples were CDI-positive via toxin culture despite failing the stool EIA test for toxin [43]. Of particular concern, they did not find any difference in mortality or CDI recurrence between EIA Tox^−^ and EIA Tox^+^ patients. As such, a failure to detect toxin in stool should be interpreted with caution, especially if patients exhibit clinical signs associated with *C. difficile* infection.

Our studies suggest that low-toxin strains exhibit a hyper-colonization phenotype compared to high-toxin strains, regardless of ribotype. Specifically, LT-027 strains elaborate a distinct surface architecture, that elicits an equally distinct serum reactivity, which may contribute to robust gut colonization and persistence. This persistence is likely not due to variations in growth rates which were similar between all RT027 strains. A distinct LT-027 strain cell surface may potentiate colonization via impacts on host-cell attachment, innate immune evasion, or other mechanisms; these are yet to be determined.

It is also possible that an altered cell surface is genetically linked to a low-toxin phenotype suggestive of a convergent set of genetic changes. The pangenomes of LT-027 and those of unrelated *C. difficile* lineages like RT106, share only one gene. This lack of inter-lineage gene-content overlap is consistent with the enormous plasticity of the *C. difficile* genome [44], and many imply evolutionary/genetic mechanisms that, on the whole, potentiate stable low-toxin and colonization phenotypes.

From a selection standpoint, low-toxin *C. difficile* strains may proliferate, or become endemic, in hospital settings (especially where there is reliance on stool toxin tests) precisely because these strains escape attention or treatment. Toxin expression is highly regulated and intricately linked to *C. difficile* metabolism [45–50] and the metabolic cost of producing TcdA and TcdB is high. There are no data to suggest a benefit to *C. difficile* producing high quantities of these large molecules. In their study of clinical *Staphylococcus aureus* strains, Laabei et al demonstrated that low-toxicity isolates had a higher propensity to cause bacteraemia than highly toxic strains [51]. Their work suggested that the high energy requirements of toxin production might exact a fitness cost on high-toxin isolates within the host. Interestingly, Hollands et al noted that a genetic switch to hypervirulence in Group A *Streptococcus* strains decreased the colonization capacity of these strains [52]. These findings are consistent with the idea that bacterial virulence is a trade-off between toxicity, relative fitness, and transmissibility.

Taken together, work by us and others indicate that CDI cases are likely being missed and underreported to an unknown degree if EIAs are the sole diagnostic approach. Though dogma suggests that less toxin will result in milder symptoms, our studies demonstrate that low-toxin-producing *C. difficile* strains potentiate fulminant disease in, and persistently colonize, rodent models. Since these strains were all recovered from symptomatic (diarrhoeal) human patients, it is imperative that EIA-discrepant CDI test results are not overlooked.

## Supplementary Material

Supplemental Material
