# Supplementary material for: Low-toxin Clostridioides difficile RT027 strains exhibit robust virulence
Source: Emerg Microbes Infect. 2022 Aug 8;11(1):1982–93. doi: 10.1080/22221751.2022.2105260 (PMC9361768; doi:10.1080/22221751.2022.2105260)
Supplement: Supplemental Material [file TEMI_A_2105260_SM9468.docx]

**Low-Toxin *Clostridioides difficile* RT027 Strains Exhibit Robust Virulence**

Farhan Anwar^1^, Bryan Angelo P. Roxas^1^, Kareem W. Shehab^2^, Neil Ampel^3^, VK Viswanathan^1,4,5^, and Gayatri Vedantam^1, 4, 5, 6^

^1^School of Animal and Comparative Biomedical Sciences, The University of Arizona, Tucson, AZ, USA, ^2^Department of Pediatrics, The University of Arizona College of Medicine, Tucson, AZ, USA, ^3^Mayo Clinic, Phoenix, AZ, ^4^Department of Immunobiology, The University of Arizona College of Medicine, Tucson, AZ, USA, ^5^BIO5 Institute for Collaborative Research, The University of Arizona, Tucson, AZ, ^6^Southern Arizona VA Healthcare System, Tucson, AZ^4^.

*Correspondence to:

Gayatri Vedantam, Ph.D.

University of Arizona

School of Animal & Comparative Biomedical Sciences

1117, E. Lowell St. Bldg. 90, Room 227

Tucson, AZ 85721

Phone: 520-626-6839

[gayatri@email.arizona.edu](mailto:gayatri@email.arizona.edu)

**Supplemental Table S1: Primers Used In this Study**

| **Primer name** |  | **Gene Target** | **Sequence** | **Ref** |
| --- | --- | --- | --- | --- |
| RIBO-F |  | 16S rDNA | 5′-GCTGGATCACCTCCTTTCTAAG | Janežič et al. *J Clin Microbiol* (2011) |
| RIBO-R |  | 23S rDNA | 5′-TGACCAGTTAAAAAGGTTTGATAGATT |  |
| B1C |  | *tcdB* | 5' - AGAAAATTTTATGAGTTTAGTTAATAGAAA | Rupnik et al. *FEMS Microbiol Lett* (2006) |
| B2N |  | *tcdB* | 5' - CAGATAATGTAGGAAGTAAGTCTATAG |  |
| A1C |  | *tcdA* | 5' - GGAGGTTTTTATGTCTTTAATATCTAAAGA |  |
| A2N |  | *tcdA* | 5' - CCCTCTGTTATTGAGGTAGTACATTTA |  |
| cdtA-F |  | *cdtA* | 5′-TGAACCTGGAAAAGGTGATG | Pituch et al. *J Med Microbiol* (2005) |
| cdtA-R |  | *cdtA* | 5′-AGGATTATTTACTGGACCATTTG |  |
| cdtB-F |  | *cdtB* | 5′-CTTAATGCAAGTAAATACTGAG |  |
| cdtB-R |  | *cdtB* | 5′-AACGGATCTCTTGCTTCAGTC |  |
| Tim2 |  | *tcdC* | 5’-GCACCTCATCACCATCTTCA | Cohen et al. *J Infect Dis* (2000) |
| Struppi2 |  | *tcdC* | 5’-TGAAGACCATGAGGAGGTCAT |  |

| **Strain** | **NCBI Accession Numbers** | | **Coverage** |
| --- | --- | --- | --- |
|  | **Biosample** | **BioProject** |  |
| GV106 | SAMN26752631 | PRJNA817265 | 630x |
| GV135 | SAMN26752621 | PRJNA817265 | 2060x |
| GV144 | SAMN26752619 | PRJNA817265 | 65x |
| GV145 | SAMN26752625 | PRJNA817265 | 142x |
| GV147 | SAMN26752628 | PRJNA817265 | 69x |
| GV148 | SAMN26752630 | PRJNA817265 | 751x |
| GV153 | SAMN26752620 | PRJNA817265 | 51x |
| GV155 | SAMN26752626 | PRJNA817265 | 142x |
| GV161 | SAMN26752624 | PRJNA817265 | 134x |
| GV163 | SAMN26752633 | PRJNA817265 | 145x |
| GV165 | SAMN26752635 | PRJNA817265 | 157x |
| GV175 | SAMN26752627 | PRJNA817265 | 120x |
| GV178 | SAMN26752623 | PRJNA817265 | 118x |
| GV183 | SAMN26752629 | PRJNA817265 | 98x |
| GV736 | SAMN26752622 | PRJNA817265 | 195x |
| GV745 | SAMN26752634 | PRJNA817265 | 120x |
| GV752 | SAMN26752632 | PRJNA817265 | 318x |

**Supplemental Table S2: GenBank Accession Numbers of LT-027 Isolates**

**Supplemental Table S3: MIC of Select Antibiotics of High- and Low-Toxin Strains**

| **MIC (µg/mL)** | **Cefotaxime** | **Rifampicin** | **Levofloxacin** | **Metronidazole** | **Vancomycin** |
| --- | --- | --- | --- | --- | --- |
| **BI-1** | >16 * | 1 | >32 * | >256 * | 3 |
| **GV106** | >16 * | 1 | >32 * | >256 * | 3 |
| **GV135** | 0.75 * | 3 | 0.75 | >256 * | 3 |
| **GV148** | >16 * | >32 * | >32 * | 1 | 4 |

Units are in µg/mL.

*Denotes that the strain is highly resistant as per CLSI breakpoints:

Cefotaxime: >16 µg/mL

Rifampicin: > 16 µg/mL

Levofloxacin: > 8 µg/mL

Metronidazole: >32 µg/mL

Vancomycin: not defined.

**Supplemental Table S4: Unique genes harbored by LT-027 strains**

| **Gene ID** | **Function** |
| --- | --- |
| fig\|1496.1250.peg.42 | FIG002813: LPPG:FO 2-phospho-L-lactate transferase like, CofD-like |
| fig\|1496.1250.peg.146 | FIG00514768: hypothetical protein |
| fig\|1496.1250.peg.155 | Anaerobic sulfite reductase subunit B |
| fig\|1496.1250.peg.164 | N-acetylmannosamine-6-phosphate 2-epimerase (EC 5.1.3.9) |
| fig\|1496.1250.peg.173 | ATPase associated with various cellular activities, AAA_5 |
| fig\|1496.1250.peg.198 | FIG00522519: hypothetical protein |
| fig\|1496.1250.peg.371 | tRNA and rRNA cytosine-C5-methylases |
| fig\|1496.1250.peg.394 | FIG00520447: hypothetical protein |
| fig\|1496.1250.peg.407 | FIG00512777: hypothetical protein |
| fig\|1496.1250.peg.408 | FIG00515422: hypothetical protein |
| fig\|1496.1250.peg.470 | Cytosol nonspecific dipeptidase (EC 3.4.13.18) |
| fig\|1496.1250.peg.661 | Osmosensitive K+ channel histidine kinase KdpD (EC 2.7.3.-) |
| fig\|1496.1250.peg.755 | Chloride channel protein |
| fig\|1496.1250.peg.845 | D-aminopeptidase dipeptide-binding protein DppA (EC 3.4.11.-)* |
| fig\|1496.1250.peg.965 | LSU ribosomal protein L13p (L13Ae) |
| fig\|1496.1250.peg.1001 | DNA topoisomerase I (EC 5.99.1.2) |
| fig\|1496.1250.peg.1050 | Translocation-enhancing protein TepA |
| fig\|1496.1250.peg.1145 | RNA polymerase sporulation specific sigma factor SigE |
| fig\|1496.1250.peg.1158 | Acyl-CoA:1-acyl-sn-glycerol-3-phosphate acyltransferase (EC 2.3.1.51) |
| fig\|1496.1250.peg.1173 | 3-dehydroquinate synthase (EC 4.2.3.4) |
| fig\|1496.1250.peg.1221 | AzlC family protein |
| fig\|1496.1250.peg.1256 | N-acetylmuramoyl-L-alanine amidase (EC 3.5.1.28)* |
| fig\|1496.1250.peg.1324 | Transcriptional regulator |
| fig\|1496.1250.peg.1335 | Hydantoinase/oxoprolinase family protein |
| fig\|1496.1250.peg.1416 | putative membrane protein (putative phage infection protein)* |
| fig\|1496.1250.peg.1419 | Rubrerythrin |
| fig\|1496.1250.peg.1455 | FIG00519734: hypothetical protein |
| fig\|1496.1250.peg.1483 | FIG00514478: hypothetical protein |
| fig\|1496.1250.peg.1580 | Peptide methionine sulfoxide reductase MsrA (EC 1.8.4.11) / Peptide methionine sulfoxide reductase MsrB (EC 1.8.4.12) |
| fig\|1496.1250.peg.1736 | V-type ATP synthase subunit D (EC 3.6.3.14) |
| fig\|1496.1250.peg.2084 | Stage III sporulation protein AB |
| fig\|1496.1250.peg.2198 | SSU ribosomal protein S20p |
| fig\|1496.1250.peg.2205 | Cell wall-binding protein* |
| fig\|1496.1250.peg.2206 | Uracil-DNA glycosylase, family 1 |
| fig\|1496.1250.peg.2337 | FIG013354: hypothetical protein |
| fig\|1496.1250.peg.2338 | UDP-N-acetylglucosamine 1-carboxyvinyltransferase (EC 2.5.1.7)* |
| fig\|1496.1250.peg.2342 | MreB-like protein (Mbl protein) |
| fig\|1496.1250.peg.2378 | hypothetical protein |
| fig\|1496.1250.peg.2379 | RNA polymerase sporulation specific sigma factor SigK |
| fig\|1496.1250.peg.2413 | Peptidoglycan N-acetylglucosamine deacetylase (EC 3.5.1.-)* |
| fig\|1496.1250.peg.2440 | ATP phosphoribosyltransferase (EC 2.4.2.17) |
| fig\|1496.1250.peg.2466 | FIG00522895: hypothetical protein |
| fig\|1496.1250.peg.2476 | FIG006036: phage encoded DNA polymerase I (EC 2.7.7.7) |
| fig\|1496.1250.peg.2527 | HNH homing endonuclease |
| fig\|1496.1250.peg.2560 | ABC transporter, permease protein* |
| fig\|1496.1250.peg.2609 | Formate dehydrogenase H (EC 1.2.1.2) @ selenocysteine-containing |
| fig\|1496.1250.peg.2611 | Response regulator |
| fig\|1496.1250.peg.2670 | Arsenic efflux pump protein* |
| fig\|1496.1250.peg.2790 | Dihydropyrimidinase (EC 3.5.2.2) @ D-hydantoinase (EC 3.5.2.2) |
| fig\|1496.1250.peg.2815 | Transcriptional regulator, MerR family |
| fig\|1496.1250.peg.2862 | Transcriptional regulator, MecI family |
| fig\|1496.1250.peg.2904 | FIG004454: RNA binding protein |
| fig\|1496.1250.peg.2992 | FIG00516716: hypothetical protein |
| fig\|1496.1250.peg.3048 | Sarcosine reductase component B beta subunit (EC 1.21.4.3) |
| fig\|1496.1250.peg.3092 | Hydrolase, HAD superfamily |
| fig\|1496.1250.peg.3097 | Membrane component of multidrug resistance system* |
| fig\|1496.1250.peg.3102 | Beta-glucoside bgl operon antiterminator, BglG family |
| fig\|1496.1250.peg.3114 | RecA regulator RecX |
| fig\|1496.1250.peg.3123 | Phosphate regulon transcriptional regulatory protein PhoB (SphR) |
| fig\|1496.1250.peg.3218 | FIG00518106: hypothetical protein |
| fig\|1496.1250.peg.3269 | Foldase protein PrsA precursor (EC 5.2.1.8) |
| fig\|1496.1250.peg.3342 | Glycerol-3-phosphate regulon repressor GlpR |
| fig\|1496.1250.peg.3462 | ATP-dependent DNA helicase UvrD/PcrA |
| fig\|1496.1250.peg.3656 | FIG001621: Zinc protease |
| fig\|1496.1250.peg.3859 | putative PTS system, IIa component |
| fig\|1496.1250.peg.3966 | Ribonucleotide reductase of class III (anaerobic), activating protein  (EC 1.97.1.4) |

**Cell-wall associated genes denoted by asterisk**


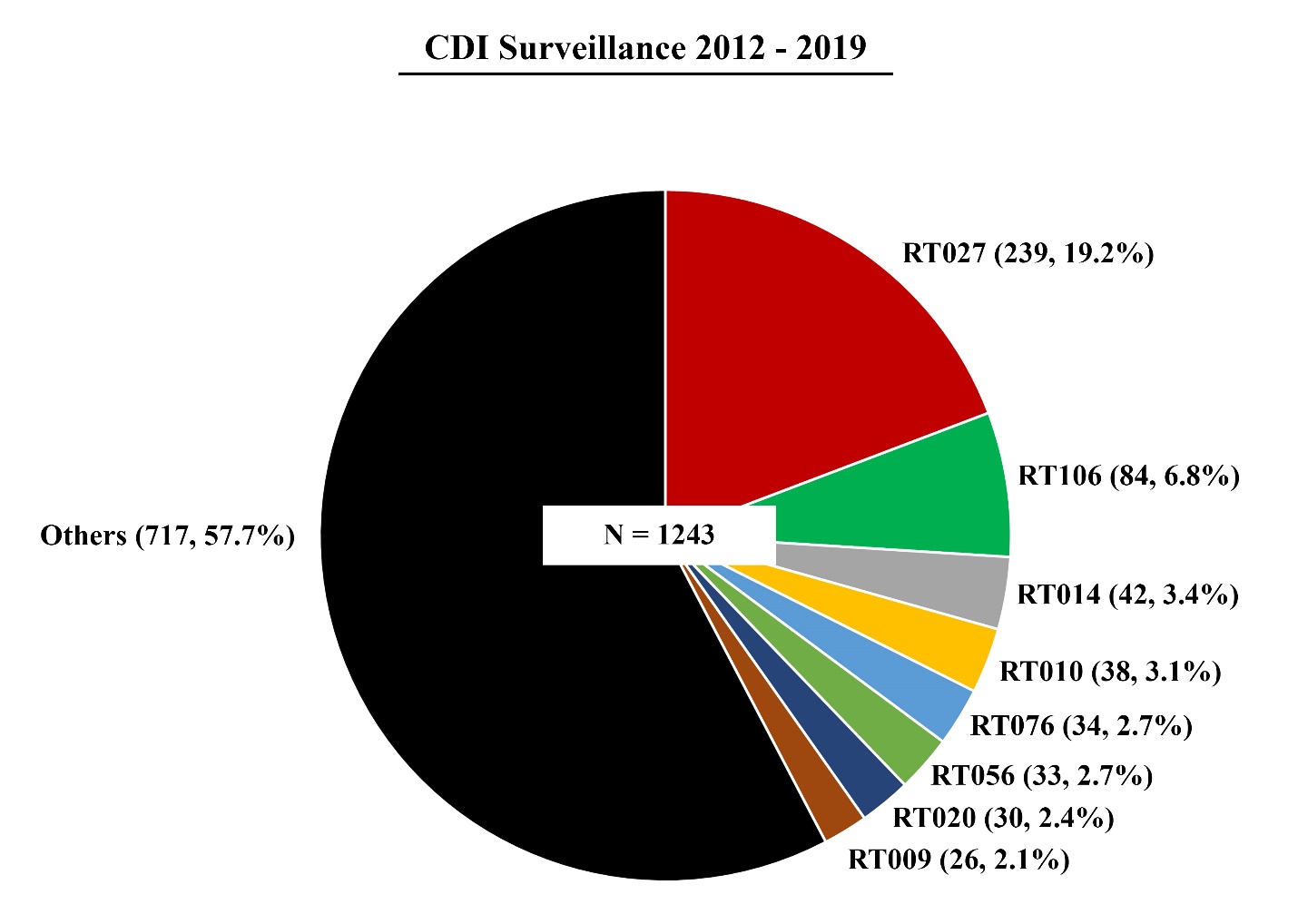


**Supplemental Figure S1: *Clostridioides difficile* Ribotype RT027 is Prevalent in Tucson-area Hospitals**

Ribotype distribution of *C. difficile* strains isolated from patient stool specimens collected from 2012-2019 (n=1243). Ribotype frequency and percent of total sample size are shown in parentheses (number of isolates, and percent of total). RT027 is the predominant ribotype. ‘Others’ refers to ribotypes that each constitute 2% or less of the total.


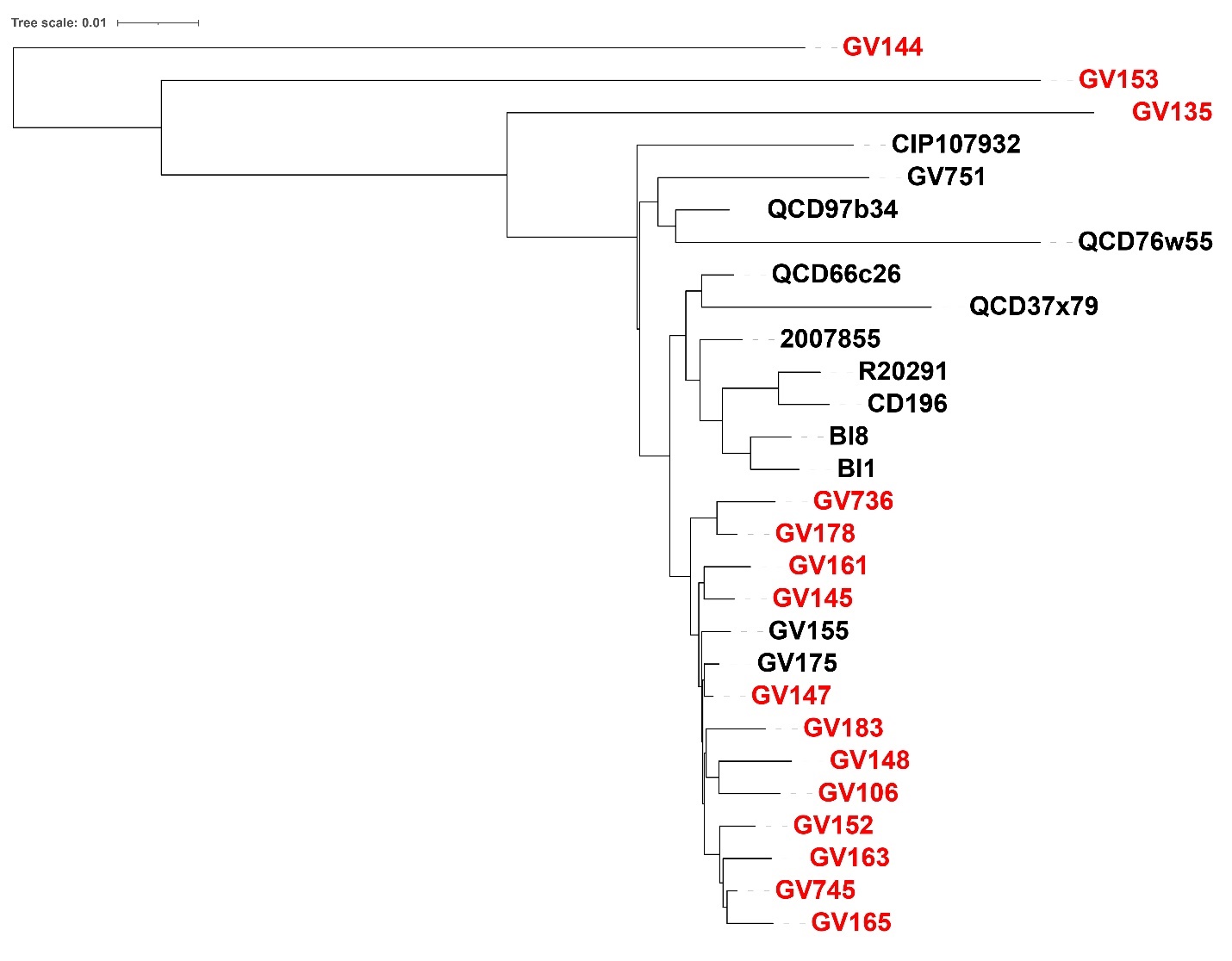


**Supplemental Figure S2: Phylogenetic Comparison of High- and Low-Toxin RT027 Strains**

Whole genome comparison between clinically derived, low-toxin producing (n = 15; red) and high toxin producing strains (n = 13; black). The high toxin strains (black) were previously published (except for GV155 and GV175). Low- and high-toxin strains cluster into distinct sub-clades. There are, at minimum, 80 single nucleotide polymorphism (SNP) differences between the most closely related low-toxin strains indicating that isolates are not identical, and, therefore, clonal.

**
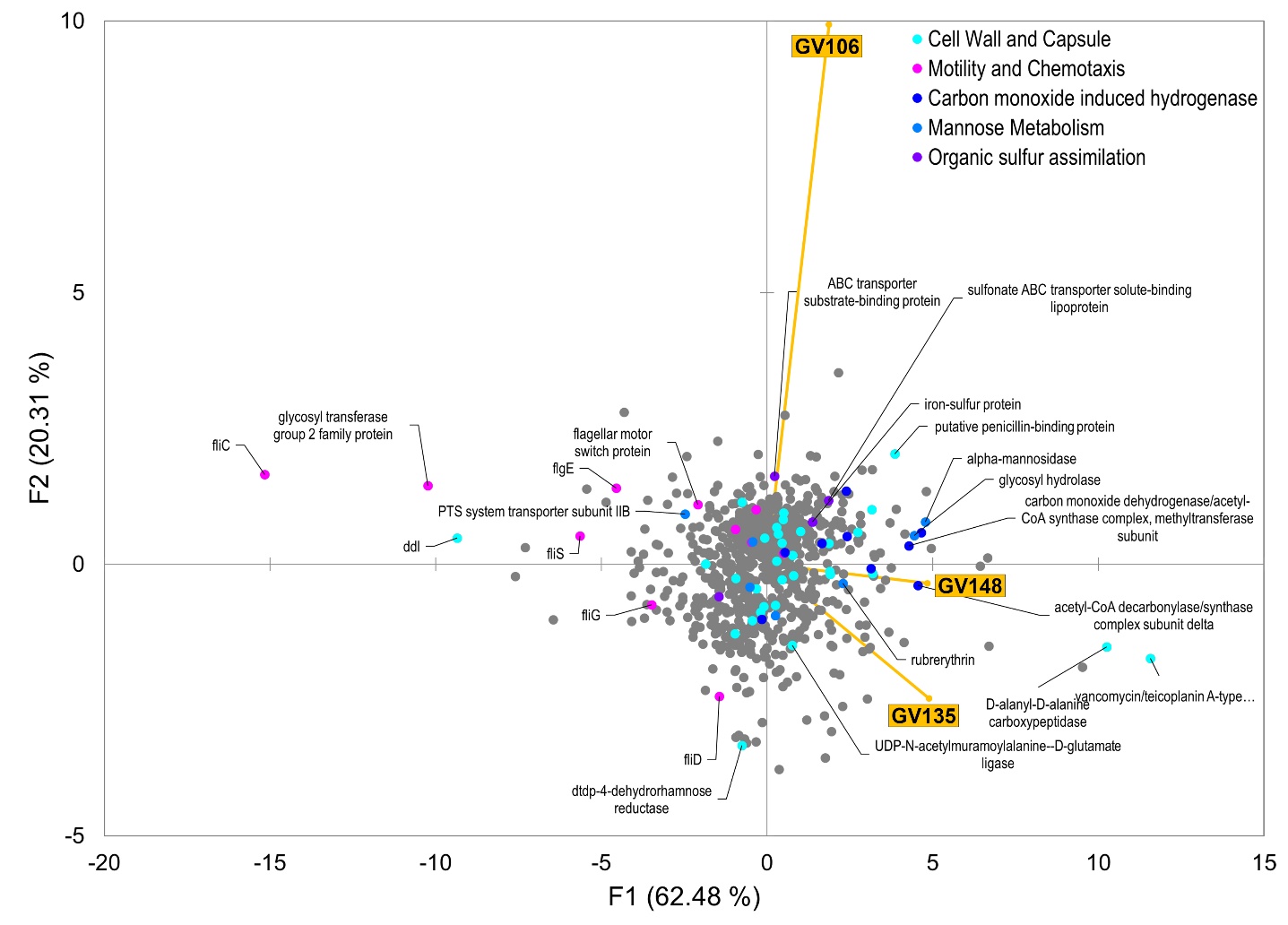
**

**Supplemental Figure S3: PCA of LT-027 protein abundances relative to BI-1.**

Protein abundances of low-toxin strains (at mid-logarithmic growth phase), were compared to the high-toxin producer BI-1. Each dot represents a protein hit; colored dots are proteins involved in selected biological processes, and which were displayed statistically significant differences in abundance compared with BI-1. Several of these proteins are predicted to be involved in cell wall and capsular biology.

**Supplemental Table S5: KEGG Subcategories of LT-027 unique genes.**

| **Gene ID** | **Function** | **KEGG Classification ID** | **KEGG Classification** | **KEGG Classification Description** | **KEGG Gene ID** | **KEGG Gene Description** |
| --- | --- | --- | --- | --- | --- | --- |
| fig\|1496.1250.peg.155 | Anaerobic sulfite reductase subunit B | ko01100 | Pathway | Metabolic pathways | K16951 | asrB; anaerobic sulfite reductase subunit B |
| fig\|1496.1250.peg.155 | Anaerobic sulfite reductase subunit B | ko01120 | Pathway | Microbial metabolism in diverse environments | K16951 | asrB; anaerobic sulfite reductase subunit B |
| fig\|1496.1250.peg.155 | Anaerobic sulfite reductase subunit B | ko00920 | Pathway | Sulfur metabolism | K16951 | asrB; anaerobic sulfite reductase subunit B |
| fig\|1496.1250.peg.164 | N-acetylmannosamine-6-phosphate 2-epimerase (EC 5.1.3.9) | ko01100 | Pathway | Metabolic pathways | K01788 | nanE; N-acylglucosamine-6-phosphate 2-epimerase [EC:5.1.3.9] |
| fig\|1496.1250.peg.164 | N-acetylmannosamine-6-phosphate 2-epimerase (EC 5.1.3.9) | ko00520 | Pathway | Amino sugar and nucleotide sugar metabolism | K01788 | nanE; N-acylglucosamine-6-phosphate 2-epimerase [EC:5.1.3.9] |
| fig\|1496.1250.peg.164 | N-acetylmannosamine-6-phosphate 2-epimerase (EC 5.1.3.9) | ko01000 | Brite | Enzymes | K01788 | nanE; N-acylglucosamine-6-phosphate 2-epimerase [EC:5.1.3.9] |
| fig\|1496.1250.peg.198 | FIG00522519: hypothetical protein | ko01100 | Pathway | Metabolic pathways | K00074 | paaH; 3-hydroxybutyryl-CoA dehydrogenase [EC:1.1.1.157] |
| fig\|1496.1250.peg.198 | FIG00522519: hypothetical protein | ko01120 | Pathway | Microbial metabolism in diverse environments | K00074 | paaH; 3-hydroxybutyryl-CoA dehydrogenase [EC:1.1.1.157] |
| fig\|1496.1250.peg.198 | FIG00522519: hypothetical protein | ko01200 | Pathway | Carbon metabolism | K00074 | paaH; 3-hydroxybutyryl-CoA dehydrogenase [EC:1.1.1.157] |
| fig\|1496.1250.peg.198 | FIG00522519: hypothetical protein | ko00362 | Pathway | Benzoate degradation | K00074 | paaH; 3-hydroxybutyryl-CoA dehydrogenase [EC:1.1.1.157] |
| fig\|1496.1250.peg.198 | FIG00522519: hypothetical protein | ko00650 | Pathway | Butanoate metabolism | K00074 | paaH; 3-hydroxybutyryl-CoA dehydrogenase [EC:1.1.1.157] |
| fig\|1496.1250.peg.198 | FIG00522519: hypothetical protein | ko00360 | Pathway | Phenylalanine metabolism | K00074 | paaH; 3-hydroxybutyryl-CoA dehydrogenase [EC:1.1.1.157] |
| fig\|1496.1250.peg.198 | FIG00522519: hypothetical protein | ko01000 | Brite | Enzymes | K00074 | paaH; 3-hydroxybutyryl-CoA dehydrogenase [EC:1.1.1.157] |
| fig\|1496.1250.peg.198 | FIG00522519: hypothetical protein | M00878 | Module | Phenylacetate degradation, phenylaxetate => acetyl-CoA/succinyl-CoA | K00074 | paaH; 3-hydroxybutyryl-CoA dehydrogenase [EC:1.1.1.157] |
| fig\|1496.1250.peg.198 | FIG00522519: hypothetical protein | ko02020 | Pathway | Two-component system | K03406 | mcp; methyl-accepting chemotaxis protein |
| fig\|1496.1250.peg.198 | FIG00522519: hypothetical protein | ko02030 | Pathway | Bacterial chemotaxis | K03406 | mcp; methyl-accepting chemotaxis protein |
| fig\|1496.1250.peg.198 | FIG00522519: hypothetical protein | ko02035 | Brite | Bacterial motility proteins | K03406 | mcp; methyl-accepting chemotaxis protein |
| fig\|1496.1250.peg.394 | FIG00520447: hypothetical protein | ko03000 | Brite | Transcription factors | K03973 | pspC; phage shock protein C |
| fig\|1496.1250.peg.394 | FIG00520447: hypothetical protein | ko02048 | Brite | Prokaryotic defense system | K03973 | pspC; phage shock protein C |
| fig\|1496.1250.peg.394 | FIG00520447: hypothetical protein | ko01000 | Brite | Enzymes | K03669 | mdoH; membrane glycosyltransferase [EC:2.4.1.-] |
| fig\|1496.1250.peg.394 | FIG00520447: hypothetical protein | ko01003 | Brite | Glycosyltransferases | K03669 | mdoH; membrane glycosyltransferase [EC:2.4.1.-] |
| fig\|1496.1250.peg.407 | FIG00512777: hypothetical protein | ko01100 | Pathway | Metabolic pathways | K00616 | E2.2.1.2; transaldolase [EC:2.2.1.2] |
| fig\|1496.1250.peg.407 | FIG00512777: hypothetical protein | ko01120 | Pathway | Microbial metabolism in diverse environments | K00616 | E2.2.1.2; transaldolase [EC:2.2.1.2] |
| fig\|1496.1250.peg.407 | FIG00512777: hypothetical protein | ko01110 | Pathway | Biosynthesis of secondary metabolites | K00616 | E2.2.1.2; transaldolase [EC:2.2.1.2] |
| fig\|1496.1250.peg.407 | FIG00512777: hypothetical protein | ko01230 | Pathway | Biosynthesis of amino acids | K00616 | E2.2.1.2; transaldolase [EC:2.2.1.2] |
| fig\|1496.1250.peg.407 | FIG00512777: hypothetical protein | ko01200 | Pathway | Carbon metabolism | K00616 | E2.2.1.2; transaldolase [EC:2.2.1.2] |
| fig\|1496.1250.peg.407 | FIG00512777: hypothetical protein | ko00030 | Pathway | Pentose phosphate pathway | K00616 | E2.2.1.2; transaldolase [EC:2.2.1.2] |
| fig\|1496.1250.peg.407 | FIG00512777: hypothetical protein | ko01000 | Brite | Enzymes | K00616 | E2.2.1.2; transaldolase [EC:2.2.1.2] |
| fig\|1496.1250.peg.407 | FIG00512777: hypothetical protein | M00007 | Module | Pentose phosphate pathway, non-oxidative phase, fructose 6P => ribose 5P | K00616 | E2.2.1.2; transaldolase [EC:2.2.1.2] |
| fig\|1496.1250.peg.407 | FIG00512777: hypothetical protein | M00004 | Module | Pentose phosphate pathway (Pentose phosphate cycle) | K00616 | E2.2.1.2; transaldolase [EC:2.2.1.2] |
| fig\|1496.1250.peg.407 | FIG00512777: hypothetical protein | ko01100 | Pathway | Metabolic pathways | K00942 | gmk; guanylate kinase [EC:2.7.4.8] |
| fig\|1496.1250.peg.407 | FIG00512777: hypothetical protein | ko01232 | Pathway | Nucleotide metabolism | K00942 | gmk; guanylate kinase [EC:2.7.4.8] |
| fig\|1496.1250.peg.407 | FIG00512777: hypothetical protein | ko00230 | Pathway | Purine metabolism | K00942 | gmk; guanylate kinase [EC:2.7.4.8] |
| fig\|1496.1250.peg.407 | FIG00512777: hypothetical protein | ko01000 | Brite | Enzymes | K00942 | gmk; guanylate kinase [EC:2.7.4.8] |
| fig\|1496.1250.peg.407 | FIG00512777: hypothetical protein | M00050 | Module | Guanine ribonucleotide biosynthesis, IMP => GDP,GTP | K00942 | gmk; guanylate kinase [EC:2.7.4.8] |
| fig\|1496.1250.peg.470 | Cytosol nonspecific dipeptidase (EC 3.4.13.18) | ko01100 | Pathway | Metabolic pathways | K01270 | pepD; dipeptidase D [EC:3.4.13.-] |
| fig\|1496.1250.peg.470 | Cytosol nonspecific dipeptidase (EC 3.4.13.18) | ko00480 | Pathway | Glutathione metabolism | K01270 | pepD; dipeptidase D [EC:3.4.13.-] |
| fig\|1496.1250.peg.470 | Cytosol nonspecific dipeptidase (EC 3.4.13.18) | ko01000 | Brite | Enzymes | K01270 | pepD; dipeptidase D [EC:3.4.13.-] |
| fig\|1496.1250.peg.470 | Cytosol nonspecific dipeptidase (EC 3.4.13.18) | ko01002 | Brite | Peptidases and inhibitors | K01270 | pepD; dipeptidase D [EC:3.4.13.-] |
| fig\|1496.1250.peg.661 | Osmosensitive K+ channel histidine kinase KdpD (EC 2.7.3.-) | ko02020 | Pathway | Two-component system | K07646 | kdpD; two-component system, OmpR family, sensor histidine kinase KdpD [EC:2.7.13.3] |
| fig\|1496.1250.peg.661 | Osmosensitive K+ channel histidine kinase KdpD (EC 2.7.3.-) | ko01000 | Brite | Enzymes | K07646 | kdpD; two-component system, OmpR family, sensor histidine kinase KdpD [EC:2.7.13.3] |
| fig\|1496.1250.peg.661 | Osmosensitive K+ channel histidine kinase KdpD (EC 2.7.3.-) | ko02022 | Brite | Two-component system | K07646 | kdpD; two-component system, OmpR family, sensor histidine kinase KdpD [EC:2.7.13.3] |
| fig\|1496.1250.peg.661 | Osmosensitive K+ channel histidine kinase KdpD (EC 2.7.3.-) | ko01001 | Brite | Protein kinases | K07646 | kdpD; two-component system, OmpR family, sensor histidine kinase KdpD [EC:2.7.13.3] |
| fig\|1496.1250.peg.845 | D-aminopeptidase dipeptide-binding protein DppA (EC 3.4.11.-) | ko01000 | Brite | Enzymes | K16203 | dppA1; D-amino peptidase [EC:3.4.11.-] |
| fig\|1496.1250.peg.845 | D-aminopeptidase dipeptide-binding protein DppA (EC 3.4.11.-) | ko01002 | Brite | Peptidases and inhibitors | K16203 | dppA1; D-amino peptidase [EC:3.4.11.-] |
| fig\|1496.1250.peg.965 | LSU ribosomal protein L13p (L13Ae) | ko03010 | Pathway | Ribosome | K02871 | RP-L13; large subunit ribosomal protein L13 |
| fig\|1496.1250.peg.965 | LSU ribosomal protein L13p (L13Ae) | ko03011 | Brite | Ribosome | K02871 | RP-L13; large subunit ribosomal protein L13 |
| fig\|1496.1250.peg.1001 | DNA topoisomerase I (EC 5.99.1.2) | ko01000 | Brite | Enzymes | K03168 | topA; DNA topoisomerase I [EC:5.6.2.1] |
| fig\|1496.1250.peg.1001 | DNA topoisomerase I (EC 5.99.1.2) | ko03400 | Brite | DNA repair and recombination proteins | K03168 | topA; DNA topoisomerase I [EC:5.6.2.1] |
| fig\|1496.1250.peg.1001 | DNA topoisomerase I (EC 5.99.1.2) | ko03032 | Brite | DNA replication proteins | K03168 | topA; DNA topoisomerase I [EC:5.6.2.1] |
| fig\|1496.1250.peg.1145 | RNA polymerase sporulation specific sigma factor SigE | ko03021 | Brite | Transcription machinery | K03091 | sigH; RNA polymerase sporulation-specific sigma factor |
| fig\|1496.1250.peg.1158 | Acyl-CoA:1-acyl-sn-glycerol-3-phosphate acyltransferase (EC 2.3.1.51) | ko01100 | Pathway | Metabolic pathways | K00655 | plsC; 1-acyl-sn-glycerol-3-phosphate acyltransferase [EC:2.3.1.51] |
| fig\|1496.1250.peg.1158 | Acyl-CoA:1-acyl-sn-glycerol-3-phosphate acyltransferase (EC 2.3.1.51) | ko01110 | Pathway | Biosynthesis of secondary metabolites | K00655 | plsC; 1-acyl-sn-glycerol-3-phosphate acyltransferase [EC:2.3.1.51] |
| fig\|1496.1250.peg.1158 | Acyl-CoA:1-acyl-sn-glycerol-3-phosphate acyltransferase (EC 2.3.1.51) | ko00561 | Pathway | Glycerolipid metabolism | K00655 | plsC; 1-acyl-sn-glycerol-3-phosphate acyltransferase [EC:2.3.1.51] |
| fig\|1496.1250.peg.1158 | Acyl-CoA:1-acyl-sn-glycerol-3-phosphate acyltransferase (EC 2.3.1.51) | ko00564 | Pathway | Glycerophospholipid metabolism | K00655 | plsC; 1-acyl-sn-glycerol-3-phosphate acyltransferase [EC:2.3.1.51] |
| fig\|1496.1250.peg.1158 | Acyl-CoA:1-acyl-sn-glycerol-3-phosphate acyltransferase (EC 2.3.1.51) | ko01000 | Brite | Enzymes | K00655 | plsC; 1-acyl-sn-glycerol-3-phosphate acyltransferase [EC:2.3.1.51] |
| fig\|1496.1250.peg.1158 | Acyl-CoA:1-acyl-sn-glycerol-3-phosphate acyltransferase (EC 2.3.1.51) | ko01004 | Brite | Lipid biosynthesis proteins | K00655 | plsC; 1-acyl-sn-glycerol-3-phosphate acyltransferase [EC:2.3.1.51] |
| fig\|1496.1250.peg.1158 | Acyl-CoA:1-acyl-sn-glycerol-3-phosphate acyltransferase (EC 2.3.1.51) | M00089 | Module | Triacylglycerol biosynthesis | K00655 | plsC; 1-acyl-sn-glycerol-3-phosphate acyltransferase [EC:2.3.1.51] |
| fig\|1496.1250.peg.1173 | 3-dehydroquinate synthase (EC 4.2.3.4) | ko01100 | Pathway | Metabolic pathways | K01735 | aroB; 3-dehydroquinate synthase [EC:4.2.3.4] |
| fig\|1496.1250.peg.1173 | 3-dehydroquinate synthase (EC 4.2.3.4) | ko01110 | Pathway | Biosynthesis of secondary metabolites | K01735 | aroB; 3-dehydroquinate synthase [EC:4.2.3.4] |
| fig\|1496.1250.peg.1173 | 3-dehydroquinate synthase (EC 4.2.3.4) | ko01230 | Pathway | Biosynthesis of amino acids | K01735 | aroB; 3-dehydroquinate synthase [EC:4.2.3.4] |
| fig\|1496.1250.peg.1173 | 3-dehydroquinate synthase (EC 4.2.3.4) | ko00400 | Pathway | Phenylalanine, tyrosine and tryptophan biosynthesis | K01735 | aroB; 3-dehydroquinate synthase [EC:4.2.3.4] |
| fig\|1496.1250.peg.1173 | 3-dehydroquinate synthase (EC 4.2.3.4) | ko01000 | Brite | Enzymes | K01735 | aroB; 3-dehydroquinate synthase [EC:4.2.3.4] |
| fig\|1496.1250.peg.1173 | 3-dehydroquinate synthase (EC 4.2.3.4) | M00022 | Module | Shikimate pathway, phosphoenolpyruvate erythrose-4P => chorismate | K01735 | aroB; 3-dehydroquinate synthase [EC:4.2.3.4] |
| fig\|1496.1250.peg.1256 | N-acetylmuramoyl-L-alanine amidase (EC 3.5.1.28) | ko01503 | Pathway | Cationic antimicrobial peptide | K01448 | amiABC; N-acetylmuramoyl-L-alanine amidase [EC:3.5.1.28] |
| fig\|1496.1250.peg.1256 | N-acetylmuramoyl-L-alanine amidase (EC 3.5.1.28) | ko01000 | Brite | Enzymes | K01448 | amiABC; N-acetylmuramoyl-L-alanine amidase [EC:3.5.1.28] |
| fig\|1496.1250.peg.1256 | N-acetylmuramoyl-L-alanine amidase (EC 3.5.1.28) | ko01011 | Brite | Peptidoglycan biosynthesis and degradation proteins | K01448 | amiABC; N-acetylmuramoyl-L-alanine amidase [EC:3.5.1.28] |
| fig\|1496.1250.peg.1256 | N-acetylmuramoyl-L-alanine amidase (EC 3.5.1.28) | ko03036 | Brite | Chromosome and associated proteins | K01448 | amiABC; N-acetylmuramoyl-L-alanine amidase [EC:3.5.1.28] |
| fig\|1496.1250.peg.1324 | Transcriptional regulator | ko03000 | Brite | Transcription factors | K07729 | putative transcriptional regulator |
| fig\|1496.1250.peg.1455 | FIG00519734: hypothetical protein | ko01000 | Brite | Enzymes | K03929 | pnbA; para-nitrobenzyl esterase [EC:3.1.1.-] |
| fig\|1496.1250.peg.1483 | FIG00514478: hypothetical protein | ko02048 | Brite | Prokaryotic defense system | K09951 | cas2; CRISPR-associated protein Cas2 |
| fig\|1496.1250.peg.1483 | FIG00514478: hypothetical protein | ko01100 | Pathway | Metabolic pathways | K02798 | cmtB; mannitol PTS system EIIA component [EC:2.7.1.197] |
| fig\|1496.1250.peg.1483 | FIG00514478: hypothetical protein | ko02060 | Pathway | Phosphotransferase system | K02798 | cmtB; mannitol PTS system EIIA component [EC:2.7.1.197] |
| fig\|1496.1250.peg.1483 | FIG00514478: hypothetical protein | ko00051 | Pathway | Fructose and mannose metabolism | K02798 | cmtB; mannitol PTS system EIIA component [EC:2.7.1.197] |
| fig\|1496.1250.peg.1483 | FIG00514478: hypothetical protein | ko01000 | Brite | Enzymes | K02798 | cmtB; mannitol PTS system EIIA component [EC:2.7.1.197] |
| fig\|1496.1250.peg.1483 | FIG00514478: hypothetical protein | ko02000 | Brite | Transporters | K02798 | cmtB; mannitol PTS system EIIA component [EC:2.7.1.197] |
| fig\|1496.1250.peg.1580 | Peptide methionine sulfoxide reductase MsrA (EC 1.8.4.11) / Peptide methionine sulfoxide reductase MsrB (EC 1.8.4.12) | ko01000 | Brite | Enzymes | K12267 | msrAB; peptide methionine sulfoxide reductase msrA/msrB [EC:1.8.4.11 1.8.4.12] |
| fig\|1496.1250.peg.1736 | V-type ATP synthase subunit D (EC 3.6.3.14) | ko01100 | Pathway | Metabolic pathways | K02120 | ATPVD; V/A-type H?transporting ATPase subunit D |
| fig\|1496.1250.peg.1736 | V-type ATP synthase subunit D (EC 3.6.3.14) | ko00190 | Pathway | Oxidative phosphorylation | K02120 | ATPVD; V/A-type H?transporting ATPase subunit D |
| fig\|1496.1250.peg.1736 | V-type ATP synthase subunit D (EC 3.6.3.14) | M00159 | Module | V/A-type ATPase, prokaryotes | K02120 | ATPVD; V/A-type H?transporting ATPase subunit D |
| fig\|1496.1250.peg.2198 | SSU ribosomal protein S20p | ko03010 | Pathway | Ribosome | K02968 | RP-S20; small subunit ribosomal protein S20 |
| fig\|1496.1250.peg.2198 | SSU ribosomal protein S20p | ko03011 | Brite | Ribosome | K02968 | RP-S20; small subunit ribosomal protein S20 |
| fig\|1496.1250.peg.2206 | Uracil-DNA glycosylase, family 1 | ko03410 | Pathway | Base excision repair | K03648 | UNG; uracil-DNA glycosylase [EC:3.2.2.27] |
| fig\|1496.1250.peg.2206 | Uracil-DNA glycosylase, family 1 | ko05340 | Pathway | Primary immunodeficiency | K03648 | UNG; uracil-DNA glycosylase [EC:3.2.2.27] |
| fig\|1496.1250.peg.2206 | Uracil-DNA glycosylase, family 1 | ko01000 | Brite | Enzymes | K03648 | UNG; uracil-DNA glycosylase [EC:3.2.2.27] |
| fig\|1496.1250.peg.2206 | Uracil-DNA glycosylase, family 1 | ko03400 | Brite | DNA repair and recombination proteins | K03648 | UNG; uracil-DNA glycosylase [EC:3.2.2.27] |
| fig\|1496.1250.peg.2338 | UDP-N-acetylglucosamine 1-carboxyvinyltransferase (EC 2.5.1.7) | ko01100 | Pathway | Metabolic pathways | K00790 | murA; UDP-N-acetylglucosamine 1-carboxyvinyltransferase [EC:2.5.1.7] |
| fig\|1496.1250.peg.2338 | UDP-N-acetylglucosamine 1-carboxyvinyltransferase (EC 2.5.1.7) | ko00520 | Pathway | Amino sugar and nucleotide sugar metabolism | K00790 | murA; UDP-N-acetylglucosamine 1-carboxyvinyltransferase [EC:2.5.1.7] |
| fig\|1496.1250.peg.2338 | UDP-N-acetylglucosamine 1-carboxyvinyltransferase (EC 2.5.1.7) | ko00550 | Pathway | Peptidoglycan biosynthesis | K00790 | murA; UDP-N-acetylglucosamine 1-carboxyvinyltransferase [EC:2.5.1.7] |
| fig\|1496.1250.peg.2338 | UDP-N-acetylglucosamine 1-carboxyvinyltransferase (EC 2.5.1.7) | ko01250 | Pathway | Biosynthesis of nucleotide sugars | K00790 | murA; UDP-N-acetylglucosamine 1-carboxyvinyltransferase [EC:2.5.1.7] |
| fig\|1496.1250.peg.2338 | UDP-N-acetylglucosamine 1-carboxyvinyltransferase (EC 2.5.1.7) | ko01000 | Brite | Enzymes | K00790 | murA; UDP-N-acetylglucosamine 1-carboxyvinyltransferase [EC:2.5.1.7] |
| fig\|1496.1250.peg.2338 | UDP-N-acetylglucosamine 1-carboxyvinyltransferase (EC 2.5.1.7) | ko01011 | Brite | Peptidoglycan biosynthesis and degradation proteins | K00790 | murA; UDP-N-acetylglucosamine 1-carboxyvinyltransferase [EC:2.5.1.7] |
| fig\|1496.1250.peg.2342 | MreB-like protein (Mbl protein) | ko02048 | Brite | Prokaryotic defense system | K03569 | mreB; rod shape-determining protein MreB and related proteins |
| fig\|1496.1250.peg.2342 | MreB-like protein (Mbl protein) | ko03036 | Brite | Chromosome and associated proteins | K03569 | mreB; rod shape-determining protein MreB and related proteins |
| fig\|1496.1250.peg.2342 | MreB-like protein (Mbl protein) | ko04812 | Brite | Cytoskeleton proteins | K03569 | mreB; rod shape-determining protein MreB and related proteins |
| fig\|1496.1250.peg.2379 | RNA polymerase sporulation specific sigma factor SigK | ko03021 | Brite | Transcription machinery | K03091 | sigH; RNA polymerase sporulation-specific sigma factor |
| fig\|1496.1250.peg.2413 | Peptidoglycan N-acetylglucosamine deacetylase (EC 3.5.1.-) | ko01000 | Brite | Enzymes | K22278 | pgdA; peptidoglycan-N-acetylglucosamine deacetylase [EC:3.5.1.104] |
| fig\|1496.1250.peg.2440 | ATP phosphoribosyltransferase (EC 2.4.2.17) | ko01100 | Pathway | Metabolic pathways | K00765 | hisG; ATP phosphoribosyltransferase [EC:2.4.2.17] |
| fig\|1496.1250.peg.2440 | ATP phosphoribosyltransferase (EC 2.4.2.17) | ko01110 | Pathway | Biosynthesis of secondary metabolites | K00765 | hisG; ATP phosphoribosyltransferase [EC:2.4.2.17] |
| fig\|1496.1250.peg.2440 | ATP phosphoribosyltransferase (EC 2.4.2.17) | ko01230 | Pathway | Biosynthesis of amino acids | K00765 | hisG; ATP phosphoribosyltransferase [EC:2.4.2.17] |
| fig\|1496.1250.peg.2440 | ATP phosphoribosyltransferase (EC 2.4.2.17) | ko00340 | Pathway | Histidine metabolism | K00765 | hisG; ATP phosphoribosyltransferase [EC:2.4.2.17] |
| fig\|1496.1250.peg.2440 | ATP phosphoribosyltransferase (EC 2.4.2.17) | ko01000 | Brite | Enzymes | K00765 | hisG; ATP phosphoribosyltransferase [EC:2.4.2.17] |
| fig\|1496.1250.peg.2440 | ATP phosphoribosyltransferase (EC 2.4.2.17) | M00026 | Module | Histidine biosynthesis, PRPP => histidine | K00765 | hisG; ATP phosphoribosyltransferase [EC:2.4.2.17] |
| fig\|1496.1250.peg.2466 | FIG00522895: hypothetical protein | ko01000 | Brite | Enzymes | K23415 | papA1; 2'-acyl-2-O-sulfo-trehalose (hydroxy)phthioceranyltransferase [EC:2.3.1.283] |
| fig\|1496.1250.peg.2476 | FIG006036: phage encoded DNA polymerase I (EC 2.7.7.7) | ko01000 | Brite | Enzymes | K02334 | dpo; DNA polymerase bacteriophage-type [EC:2.7.7.7] |
| fig\|1496.1250.peg.2560 | ABC transporter, permease protein | ko02000 | Brite | Transporters | K25155 | evrB; viologen exporter family transport system permease protein |
| fig\|1496.1250.peg.2609 | Formate dehydrogenase H (EC 1.2.1.2) @ selenocysteine-containing | ko01100 | Pathway | Metabolic pathways | K22015 | fdhF; formate dehydrogenase (hydrogenase) [EC:1.17.98.4 1.17.98.-] |
| fig\|1496.1250.peg.2609 | Formate dehydrogenase H (EC 1.2.1.2) @ selenocysteine-containing | ko01120 | Pathway | Microbial metabolism in diverse environments | K22015 | fdhF; formate dehydrogenase (hydrogenase) [EC:1.17.98.4 1.17.98.-] |
| fig\|1496.1250.peg.2609 | Formate dehydrogenase H (EC 1.2.1.2) @ selenocysteine-containing | ko01200 | Pathway | Carbon metabolism | K22015 | fdhF; formate dehydrogenase (hydrogenase) [EC:1.17.98.4 1.17.98.-] |
| fig\|1496.1250.peg.2609 | Formate dehydrogenase H (EC 1.2.1.2) @ selenocysteine-containing | ko00720 | Pathway | Carbon fixation pathways in prokaryotes | K22015 | fdhF; formate dehydrogenase (hydrogenase) [EC:1.17.98.4 1.17.98.-] |
| fig\|1496.1250.peg.2609 | Formate dehydrogenase H (EC 1.2.1.2) @ selenocysteine-containing | ko00680 | Pathway | Methane metabolism | K22015 | fdhF; formate dehydrogenase (hydrogenase) [EC:1.17.98.4 1.17.98.-] |
| fig\|1496.1250.peg.2609 | Formate dehydrogenase H (EC 1.2.1.2) @ selenocysteine-containing | ko01000 | Brite | Enzymes | K22015 | fdhF; formate dehydrogenase (hydrogenase) [EC:1.17.98.4 1.17.98.-] |
| fig\|1496.1250.peg.2609 | Formate dehydrogenase H (EC 1.2.1.2) @ selenocysteine-containing | M00377 | Module | Reductive acetyl-CoA pathway (Wood-Ljungdahl pathway) | K22015 | fdhF; formate dehydrogenase (hydrogenase) [EC:1.17.98.4 1.17.98.-] |
| fig\|1496.1250.peg.2611 | Response regulator | ko02020 | Pathway | Two-component system | K20488 | nisR; two-component system, OmpR family, lantibiotic biosynthesis response regulator NisR/SpaR |
| fig\|1496.1250.peg.2611 | Response regulator | ko02024 | Pathway | Quorum sensing | K20488 | nisR; two-component system, OmpR family, lantibiotic biosynthesis response regulator NisR/SpaR |
| fig\|1496.1250.peg.2611 | Response regulator | ko02022 | Brite | Two-component system | K20488 | nisR; two-component system, OmpR family, lantibiotic biosynthesis response regulator NisR/SpaR |
| fig\|1496.1250.peg.2790 | Dihydropyrimidinase (EC 3.5.2.2) @ D-hydantoinase (EC 3.5.2.2) | ko01100 | Pathway | Metabolic pathways | K01464 | DPYS; dihydropyrimidinase [EC:3.5.2.2] |
| fig\|1496.1250.peg.2790 | Dihydropyrimidinase (EC 3.5.2.2) @ D-hydantoinase (EC 3.5.2.2) | ko00983 | Pathway | Drug metabolism - other enzymes | K01464 | DPYS; dihydropyrimidinase [EC:3.5.2.2] |
| fig\|1496.1250.peg.2790 | Dihydropyrimidinase (EC 3.5.2.2) @ D-hydantoinase (EC 3.5.2.2) | ko00240 | Pathway | Pyrimidine metabolism | K01464 | DPYS; dihydropyrimidinase [EC:3.5.2.2] |
| fig\|1496.1250.peg.2790 | Dihydropyrimidinase (EC 3.5.2.2) @ D-hydantoinase (EC 3.5.2.2) | ko00410 | Pathway | beta-Alanine metabolism | K01464 | DPYS; dihydropyrimidinase [EC:3.5.2.2] |
| fig\|1496.1250.peg.2790 | Dihydropyrimidinase (EC 3.5.2.2) @ D-hydantoinase (EC 3.5.2.2) | ko00770 | Pathway | Pantothenate and CoA biosynthesis | K01464 | DPYS; dihydropyrimidinase [EC:3.5.2.2] |
| fig\|1496.1250.peg.2790 | Dihydropyrimidinase (EC 3.5.2.2) @ D-hydantoinase (EC 3.5.2.2) | ko01000 | Brite | Enzymes | K01464 | DPYS; dihydropyrimidinase [EC:3.5.2.2] |
| fig\|1496.1250.peg.2790 | Dihydropyrimidinase (EC 3.5.2.2) @ D-hydantoinase (EC 3.5.2.2) | ko04147 | Brite | Exosome | K01464 | DPYS; dihydropyrimidinase [EC:3.5.2.2] |
| fig\|1496.1250.peg.2790 | Dihydropyrimidinase (EC 3.5.2.2) @ D-hydantoinase (EC 3.5.2.2) | M00046 | Module | Pyrimidine degradation, uracil => beta-alanine, thymine => 3-aminoisobutanoate | K01464 | DPYS; dihydropyrimidinase [EC:3.5.2.2] |
| fig\|1496.1250.peg.2862 | Transcriptional regulator, MecI family | ko01501 | Pathway | beta-Lactam resistance | K02171 | blaI; BlaI family transcriptional regulator, penicillinase repressor |
| fig\|1496.1250.peg.2862 | Transcriptional regulator, MecI family | ko03000 | Brite | Transcription factors | K02171 | blaI; BlaI family transcriptional regulator, penicillinase repressor |
| fig\|1496.1250.peg.2862 | Transcriptional regulator, MecI family | ko01504 | Brite | Antimicrobial resistance genes | K02171 | blaI; BlaI family transcriptional regulator, penicillinase repressor |
| fig\|1496.1250.peg.2862 | Transcriptional regulator, MecI family | M00627 | Module | beta-Lactam resistance, Bla system | K02171 | blaI; BlaI family transcriptional regulator, penicillinase repressor |
| fig\|1496.1250.peg.2904 | FIG004454: RNA binding protein / RNA-binding protein YhbY | ko03009 | Brite | Ribosome biogenesis | K07574 | yhbY; RNA-binding protein |
| fig\|1496.1250.peg.2992 | FIG00516716: hypothetical protein | ko01100 | Pathway | Metabolic pathways | K00763 | pncB; nicotinate phosphoribosyltransferase [EC:6.3.4.21] |
| fig\|1496.1250.peg.2992 | FIG00516716: hypothetical protein | ko01240 | Pathway | Biosynthesis of cofactors | K00763 | pncB; nicotinate phosphoribosyltransferase [EC:6.3.4.21] |
| fig\|1496.1250.peg.2992 | FIG00516716: hypothetical protein | ko00760 | Pathway | Nicotinate and nicotinamide metabolism | K00763 | pncB; nicotinate phosphoribosyltransferase [EC:6.3.4.21] |
| fig\|1496.1250.peg.2992 | FIG00516716: hypothetical protein | ko01000 | Brite | Enzymes | K00763 | pncB; nicotinate phosphoribosyltransferase [EC:6.3.4.21] |
| fig\|1496.1250.peg.2992 | FIG00516716: hypothetical protein | ko02010 | Pathway | ABC transporters | K05845 | opuC; osmoprotectant transport system substrate-binding protein |
| fig\|1496.1250.peg.2992 | FIG00516716: hypothetical protein | ko02000 | Brite | Transporters | K05845 | opuC; osmoprotectant transport system substrate-binding protein |
| fig\|1496.1250.peg.3048 | Sarcosine reductase component B beta subunit (EC 1.21.4.3) | ko01000 | Brite | Enzymes | K10672 | grdB; glycine reductase complex component B subunit gamma [EC:1.21.4.2] |
| fig\|1496.1250.peg.3102 | Beta-glucoside bgl operon antiterminator, BglG family | ko03000 | Brite | Transcription factors | K03488 | licT; beta-glucoside operon transcriptional antiterminator |
| fig\|1496.1250.peg.3114 | RecA regulator RecX | ko03400 | Brite | DNA repair and recombination proteins | K03565 | recX; regulatory protein |
| fig\|1496.1250.peg.3123 | Phosphate regulon transcriptional regulatory protein PhoB (SphR) | ko02020 | Pathway | Two-component system | K07776 | regX3; two-component system, OmpR family, response regulator RegX3 |
| fig\|1496.1250.peg.3123 | Phosphate regulon transcriptional regulatory protein PhoB (SphR) | ko02022 | Brite | Two-component system | K07776 | regX3; two-component system, OmpR family, response regulator RegX3 |
| fig\|1496.1250.peg.3269 | Foldase protein PrsA precursor (EC 5.2.1.8) | ko01000 | Brite | Enzymes | K07533 | prsA; foldase protein PrsA [EC:5.2.1.8] |
| fig\|1496.1250.peg.3269 | Foldase protein PrsA precursor (EC 5.2.1.8) | ko03110 | Brite | Chaperones and folding catalysts | K07533 | prsA; foldase protein PrsA [EC:5.2.1.8] |
| fig\|1496.1250.peg.3342 | Glycerol-3-phosphate regulon repressor GlpR | ko03000 | Brite | Transcription factors | K03436 | fruR2; DeoR family transcriptional regulator, fructose operon transcriptional repressor |
| fig\|1496.1250.peg.3462 | ATP-dependent DNA helicase UvrD/PcrA | ko03430 | Pathway | Mismatch repair | K03657 | uvrD; ATP-dependent DNA helicase UvrD/PcrA [EC:5.6.2.4] |
| fig\|1496.1250.peg.3462 | ATP-dependent DNA helicase UvrD/PcrA | ko03420 | Pathway | Nucleotide excision repair | K03657 | uvrD; ATP-dependent DNA helicase UvrD/PcrA [EC:5.6.2.4] |
| fig\|1496.1250.peg.3462 | ATP-dependent DNA helicase UvrD/PcrA | ko01000 | Brite | Enzymes | K03657 | uvrD; ATP-dependent DNA helicase UvrD/PcrA [EC:5.6.2.4] |
| fig\|1496.1250.peg.3462 | ATP-dependent DNA helicase UvrD/PcrA | ko03400 | Brite | DNA repair and recombination proteins | K03657 | uvrD; ATP-dependent DNA helicase UvrD/PcrA [EC:5.6.2.4] |
| fig\|1496.1250.peg.3859 | putative PTS system, IIa component | ko01120 | Pathway | Microbial metabolism in diverse environments | K17464 | dgaA; D-glucosaminate PTS system EIIA component [EC:2.7.1.203] |
| fig\|1496.1250.peg.3859 | putative PTS system, IIa component | ko00030 | Pathway | Pentose phosphate pathway | K17464 | dgaA; D-glucosaminate PTS system EIIA component [EC:2.7.1.203] |
| fig\|1496.1250.peg.3859 | putative PTS system, IIa component | ko02060 | Pathway | Phosphotransferase system | K17464 | dgaA; D-glucosaminate PTS system EIIA component [EC:2.7.1.203] |
| fig\|1496.1250.peg.3859 | putative PTS system, IIa component | ko01000 | Brite | Enzymes | K17464 | dgaA; D-glucosaminate PTS system EIIA component [EC:2.7.1.203] |
| fig\|1496.1250.peg.3859 | putative PTS system, IIa component | ko02000 | Brite | Transporters | K17464 | dgaA; D-glucosaminate PTS system EIIA component [EC:2.7.1.203] |
| fig\|1496.1250.peg.3966 | Ribonucleotide reductase of class III (anaerobic), activating protein (EC 1.97.1.4) | ko01000 | Brite | Enzymes | K04068 | nrdG; anaerobic ribonucleoside-triphosphate reductase activating protein [EC:1.97.1.4] |

**Supplemental Table S6: Statistically significant alterations in protein abundances on GV148 compared to BI-1**

| **Locus** | **Locus ID** | **Strain** | **Name** | **GV148:BI-1** | |
| --- | --- | --- | --- | --- | --- |
|  |  |  |  | **Fold Change**  **(Log2 Ratio)** | **P-value (-Log10)** |
| CDBI1_08005 | CDBI1_08005 | Peptoclostridium difficile BI1 | vancomycin/teicoplanin A-type resistance protein  [D-alanine-D-serine Ligase] | ***2.950*** | ***4.074*** |
| CDBI1_11295 | CDBI1_11295 | Peptoclostridium difficile BI1 | NAD-dependent 4-hydroxybutyrate dehydrogenase | ***2.432*** | ***1.452*** |
| CDBI1_08010 | CDBI1_08010 | Peptoclostridium difficile BI1 | D-alanyl-D-alanine carboxypeptidase | ***2.285*** | ***3.328*** |
| CDBI1_02690 | CDBI1_02690 | Peptoclostridium difficile BI1 | NADP-dependent glyceraldehyde-3-phosphate dehydrogenase | ***2.166*** | ***2.014*** |
| CDBI1_13615 | CDBI1_13615 | Peptoclostridium difficile BI1 | S-layer precursor protein  [Surface layer protein A] | ***1.847*** | ***6.220*** |
| obgE | CDBI1_05225 | Peptoclostridium difficile BI1 | GTPase CgtA | ***1.355*** | ***2.353*** |
| CDBI1_07840 | CDBI1_07840 | Peptoclostridium difficile BI1 | putative O-acetylserine sulfhydrylase | ***1.076*** | ***2.863*** |
| CDBI1_13635 | CDBI1_13635 | Peptoclostridium difficile BI1 | hypothetical protein CDBI1_13635 [Calcium-binding adhesion protein] | ***1.010*** | ***1.675*** |
| CDBI1_01045 | CDBI1_01045 | Peptoclostridium difficile BI1 | chaperonin GroEL | 0.718 | ***1.517*** |
| CDBI1_05845 | CDBI1_05845 | Peptoclostridium difficile BI1 | cysteine desulfurase | 0.571 | ***1.398*** |
| CDBI1_11245 | CDBI1_11245 | Peptoclostridium difficile BI1 | sigma-54 interacting transcription antiterminator | 0.425 | ***1.398*** |
| CDBI1_04765 | CDBI1_04765 | Peptoclostridium difficile BI1 | butyryl-CoA dehydrogenase | 0.345 | ***1.333*** |
| CDBI1_14240 | CDBI1_14240 | Peptoclostridium difficile BI1 | bifunctional acetaldehyde-CoA/alcohol dehydrogenase | 0.093 | ***7.116*** |
| CDBI1_00265 | CDBI1_00265 | Peptoclostridium difficile BI1 | prolyl-tRNA synthetase | -0.332 | ***1.595*** |
| thrS | CDBI1_02630 | Peptoclostridium difficile BI1 | threonyl-tRNA synthetase | -0.452 | ***1.564*** |
| CDBI1_10630 | CDBI1_10630 | Peptoclostridium difficile BI1 | cyclomaltodextrinase | -0.465 | ***1.786*** |
| CDBI1_11470 | CDBI1_11470 | Peptoclostridium difficile BI1 | quinolinate synthetase | -0.651 | ***1.705*** |
| rplB | CDBI1_00425 | Peptoclostridium difficile BI1 | 50S ribosomal protein L2 | -0.731 | ***1.678*** |
| CDBI1_17325 | CDBI1_17325 | Peptoclostridium difficile BI1 | methionyl-tRNA synthetase | ***-1.023*** | ***1.520*** |
| prdB | CDR20291_3101 | Peptoclostridium difficile R20291 | proline reductase | ***-1.050*** | ***1.560*** |
| CDBI1_03635 | CDBI1_03635 | Peptoclostridium difficile BI1 | formate acetyltransferase | ***-1.116*** | ***6.190*** |
| guaA | CDBI1_01065 | Peptoclostridium difficile BI1 | GMP synthase | ***-1.342*** | ***1.795*** |
| CDBI1_04210 | CDBI1_04210 | Peptoclostridium difficile BI1 | ABC transporter substrate-binding protein | ***-3.681*** | ***2.001*** |
| CDBI1_13585 | CDBI1_13585 | Peptoclostridium difficile BI1 | cell surface-associated cysteine protease [Cwp84] | ***-3.681*** | ***2.001*** |

Red indicates high abundance; green indicates less abundance. Increasing fold changes are reported as brighter color.

Fold changes reported in Log2 values.

P-values reported as -log10 values; a P value higher than 2 denotes very high significance.

**Supplemental Table S7: Statistically significant alterations in protein abundances of GV135 compared to BI-1.**

| **Locus** | **Locus ID** | **Strain** | **Name** | **GV135:BI-1** | |
| --- | --- | --- | --- | --- | --- |
|  |  |  |  | **Fold Change**  **(Log2 Ratio)** | **P-value (-Log10)** |
| CDBI1_08005 | CDBI1_08005 | Peptoclostridium difficile BI1 | vancomycin/teicoplanin A-type resistance protein [D-alanyl-D-serine ligase] | ***3.030*** | ***2.495*** |
| CDBI1_08010 | CDBI1_08010 | Peptoclostridium difficile BI1 | D-alanyl-D-alanine carboxypeptidase | ***2.339*** | ***4.292*** |
| CDBI1_11295 | CDBI1_11295 | Peptoclostridium difficile BI1 | NAD-dependent 4-hydroxybutyrate dehydrogenase | ***2.033*** | ***1.437*** |
| obgE | CDBI1_05225 | Peptoclostridium difficile BI1 | GTPase CgtA | ***1.595*** | ***2.361*** |
| CDBI1_01045 | CDBI1_01045 | Peptoclostridium difficile BI1 | chaperonin GroEL | ***1.143*** | ***2.314*** |
| CDBI1_07840 | CDBI1_07840 | Peptoclostridium difficile BI1 | putative O-acetylserine sulfhydrylase | **0.704** | ***2.829*** |
| CDBI1_05845 | CDBI1_05845 | Peptoclostridium difficile BI1 | cysteine desulfurase | **0.611** | ***1.819*** |
| cysS | CDBI1_00275 | Peptoclostridium difficile BI1 | cysteinyl-tRNA synthetase | **0.611** | ***1.333*** |
| CDBI1_13280 | CDBI1_13280 | Peptoclostridium difficile BI1 | putative serine hydroxymethyltransferase | **0.558** | ***1.429*** |
| CDBI1_13425 | CDBI1_13425 | Peptoclostridium difficile BI1 | phosphoenolpyruvate-protein phosphotransferase | **0.505** | ***2.426*** |
| dnaK | CDBI1_11965 | Peptoclostridium difficile BI1 | molecular chaperone DnaK | **0.306** | ***1.766*** |
| CDBI1_14240 | CDBI1_14240 | Peptoclostridium difficile BI1 | bifunctional acetaldehyde-CoA/alcohol dehydrogenase | **0.120** | ***4.820*** |
| CDBI1_15490 | CDBI1_15490 | Peptoclostridium difficile BI1 | phosphoglyceromutase | **-0.532** | ***1.320*** |
| CDBI1_09460 | CDBI1_09460 | Peptoclostridium difficile BI1 | hypothetical protein CDBI1_09460 | **-0.558** | ***1.840*** |
| CDBI1_03470 | CDBI1_03470 | Peptoclostridium difficile BI1 | acetyl-CoA decarbonylase/synthase complex subunit gamma | **-0.651** | ***1.684*** |
| CDBI1_03635 | CDBI1_03635 | Peptoclostridium difficile BI1 | formate acetyltransferase | **-0.704** | ***1.407*** |
| prfA | CDBI1_17040 | Peptoclostridium difficile BI1 | peptide chain release factor 1 | ***-0.917*** | ***1.308*** |
| CDBI1_15485 | CDBI1_15485 | Peptoclostridium difficile BI1 | enolase | ***-1.621*** | ***2.186*** |
| CDBI1_13595 | CDBI1_13595 | Peptoclostridium difficile BI1 | cell surface protein [Cell wall binding repeat-containing protein] | ***-2.684*** | ***1.858*** |
| CDBI1_04210 | CDBI1_04210 | Peptoclostridium difficile BI1 | ABC transporter substrate-binding protein | ***-4.292*** | ***2.180*** |
| CDBI1_13585 | CDBI1_13585 | Peptoclostridium difficile BI1 | cell surface-associated cysteine protease [Cell wall binding protein Cwp84] | ***-4.292*** | ***2.180*** |

Red indicates high abundance; green indicates less abundance. Increasing fold changes are reported as brighter color.

Fold changes reported in Log2 values.

P-values reported as -log10 values; a P value higher than 2 denotes very high significance.

**Supplemental Table S8: Statistically significant alterations in protein abundances of GV106 compared to BI-1.**

| **Locus** | **Locus ID** | **Strain** | **Name** | **GV106:BI-1** | |  |
| --- | --- | --- | --- | --- | --- | --- |
|  |  |  |  | **Fold Change**  **(Log2 Ratio)** | **P-value (-Log10)** | |
| CDBI1_11245 | CDBI1_11245 | Peptoclostridium difficile BI1 | sigma-54 interacting transcription antiterminator | ***0.997*** | ***1.549*** |  |
| addA | QO7_0989 | Peptoclostridium difficile F314 | helicase-exonuclease AddAB, AddA subunit | 0.385 | ***2.995*** |  |
| CDBI1_15555 | CDBI1_15555 | Peptoclostridium difficile BI1 | diaminopropionate ammonia-lyase | 0.319 | ***2.891*** |  |
| CDBI1_09370 | CDBI1_09370 | Peptoclostridium difficile BI1 | acetyl-coenzyme A carboxylase carboxyl transferase subunit alpha | -0.080 | ***1.495*** |  |
| CDBI1_12720 | CDBI1_12720 | Peptoclostridium difficile BI1 | cell-division initiation protein | -0.146 | ***1.480*** |  |
| CDBI1_16555 | CDBI1_16555 | Peptoclostridium difficile BI1 | HPr kinase/phosphorylase | -0.372 | ***2.049*** |  |
| CDBI1_01350 | CDBI1_01350 | Peptoclostridium difficile BI1 | flagellum-specific ATP synthase | -0.598 | ***2.116*** |  |
| CDBI1_17110 | CDBI1_17110 | Peptoclostridium difficile BI1 | bifunctional tetrapyrrole (Corrin/Porphyrin) methylase/nucleoside triphosphate pyrophosphohydrolase | -0.625 | ***1.538*** |  |
| CDBI1_13605 | CDBI1_13605 | Peptoclostridium difficile BI1 | S-layer protein | -0.691 | ***1.679*** |  |
| CDBI1_11885 | CDBI1_11885 | Peptoclostridium difficile BI1 | tRNA binding protein | ***-0.877*** | ***1.483*** |  |
| CDBI1_15775 | CDBI1_15775 | Peptoclostridium difficile BI1 | amidohydrolase | ***-1.143*** | ***2.345*** |  |
| CDBI1_17610 | CDBI1_17610 | Peptoclostridium difficile BI1 | hypothetical protein CDBI1_17610 | ***-1.342*** | ***1.918*** |  |
| rpsE | CDBI1_00495 | Peptoclostridium difficile BI1 | 30S ribosomal protein S5 | ***-1.541*** | ***1.718*** |  |
| CDBI1_12555 | CDBI1_12555 | Peptoclostridium difficile BI1 | methionyl-tRNA formyltransferase | ***-1.568*** | ***1.433*** |  |
| rpoB | CDBI1_00375 | Peptoclostridium difficile BI1 | DNA-directed RNA polymerase subunit beta | ***-1.688*** | ***1.428*** |  |
| CDBI1_04460 | CDBI1_04460 | Peptoclostridium difficile BI1 | hypothetical protein CDBI1_04460 | ***-1.727*** | ***1.315*** |  |
| CDBI1_00070 | CDBI1_00070 | Peptoclostridium difficile BI1 | seryl-tRNA synthetase | ***-1.754*** | ***1.787*** |  |
| CDBI1_13170 | CDBI1_13170 | Peptoclostridium difficile BI1 | amidohydrolase | ***-1.781*** | ***1.860*** |  |
| ileS | CDBI1_12715 | Peptoclostridium difficile BI1 | isoleucyl-tRNA synthetase | ***-1.847*** | ***1.563*** |  |
| CDBI1_01980 | CDBI1_01980 | Peptoclostridium difficile BI1 | putative fructose-bisphosphate aldolase | ***-1.953*** | ***3.021*** |  |
| tpiA | CDBI1_15495 | Peptoclostridium difficile BI1 | triosephosphate isomerase | ***-2.020*** | ***1.346*** |  |
| tsf | CDBI1_10370 | Peptoclostridium difficile BI1 | elongation factor Ts | ***-2.073*** | ***1.371*** |  |
| CDBI1_11280 | CDBI1_11280 | Peptoclostridium difficile BI1 | inosine 5-monophosphate dehydrogenase | ***-2.073*** | ***1.315*** |  |
| obgE | CDBI1_05225 | Peptoclostridium difficile BI1 | GTPase CgtA | ***-2.126*** | ***1.374*** |  |
| CDBI1_14055 | CDBI1_14055 | Peptoclostridium difficile BI1 | regulatory protease | ***-2.192*** | ***1.346*** |  |
| CDBI1_16190 | CDBI1_16190 | Peptoclostridium difficile BI1 | ATP-dependent protease La | ***-2.219*** | ***1.801*** |  |
| CDBI1_03635 | CDBI1_03635 | Peptoclostridium difficile BI1 | formate acetyltransferase | ***-2.272*** | ***1.401*** |  |
| CDBI1_08180 | CDBI1_08180 | Peptoclostridium difficile BI1 | guanine deaminase | ***-2.285*** | ***1.840*** |  |
| CDBI1_16485 | CDBI1_16485 | Peptoclostridium difficile BI1 | 6-phosphofructokinase | ***-2.299*** | ***1.518*** |  |
| CDBI1_11970 | CDBI1_11970 | Peptoclostridium difficile BI1 | heat shock protein | ***-2.312*** | ***1.600*** |  |
| QUI_3846 | QUI_3846 | Peptoclostridium difficile P59 | vitamin B12 dependent methionine synthase, activation domain protein | ***-2.352*** | ***1.338*** |  |
| CDBI1_13615 | CDBI1_13615 | Peptoclostridium difficile BI1 | S-layer precursor protein  [Surface layer protein A] | ***-2.379*** | ***1.967*** |  |
| CDBI1_02215 | CDBI1_02215 | Peptoclostridium difficile BI1 | MarR family transcriptional regulator | ***-2.432*** | ***1.883*** |  |
| CDBI1_13610 | CDBI1_13610 | Peptoclostridium difficile BI1 | preprotein translocase subunit SecA | ***-2.432*** | ***1.754*** |  |
| CDBI1_12685 | CDBI1_12685 | Peptoclostridium difficile BI1 | peptidase | ***-2.525*** | ***2.259*** |  |
| CDBI1_09460 | CDBI1_09460 | Peptoclostridium difficile BI1 | hypothetical protein CDBI1_09460 [Coenzyme F420-0:L-glutamate ligase] | ***-2.631*** | ***2.625*** |  |
| CDBI1_01960 | CDBI1_01960 | Peptoclostridium difficile BI1 | acyl-CoA dehydrogenase family protein | ***-2.671*** | ***2.345*** |  |
| rplD | CDBI1_00415 | Peptoclostridium difficile BI1 | 50S ribosomal protein L4 | ***-2.697*** | ***1.373*** |  |
| cysS | CDBI1_00275 | Peptoclostridium difficile BI1 | cysteinyl-tRNA synthetase | ***-2.724*** | ***1.781*** |  |
| CDBI1_10585 | CDBI1_10585 | Peptoclostridium difficile BI1 | aromatic compounds hydrolase | ***-2.737*** | ***1.562*** |  |
| CDBI1_13425 | CDBI1_13425 | Peptoclostridium difficile BI1 | phosphoenolpyruvate-protein phosphotransferase | ***-2.777*** | ***1.987*** |  |
| CDBI1_01940 | CDBI1_01940 | Peptoclostridium difficile BI1 | isocaprenoyl-CoA:2-hydroxyisocaproate CoA-transferase | ***-2.804*** | ***1.342*** |  |
| aspS | CDBI1_13350 | Peptoclostridium difficile BI1 | aspartyl-tRNA synthetase | ***-2.870*** | ***1.349*** |  |
| CDBI1_01965 | CDBI1_01965 | Peptoclostridium difficile BI1 | electron transfer flavoprotein subunit beta | ***-2.883*** | ***2.352*** |  |
| CDBI1_01770 | CDBI1_01770 | Peptoclostridium difficile BI1 | putative aminopeptidase 2 | ***-2.883*** | ***1.643*** |  |
| CDBI1_13210 | CDBI1_13210 | Peptoclostridium difficile BI1 | cell surface protein [Cell wall binding protein Cwp22] | ***-2.897*** | ***1.349*** |  |
| CDBI1_13280 | CDBI1_13280 | Peptoclostridium difficile BI1 | putative serine hydroxymethyltransferase | ***-2.937*** | ***2.908*** |  |
| CDBI1_04210 | CDBI1_04210 | Peptoclostridium difficile BI1 | ABC transporter substrate-binding protein | ***-2.937*** | ***1.846*** |  |
| CDBI1_13585 | CDBI1_13585 | Peptoclostridium difficile BI1 | cell surface-associated cysteine protease [Cell wall binding protein Cwp84] | ***-2.937*** | ***1.846*** |  |
| CDBI1_16105 | CDBI1_16105 | Peptoclostridium difficile BI1 | glucose-6-phosphate isomerase | ***-3.030*** | ***2.258*** |  |
| CDBI1_11210 | CDBI1_11210 | Peptoclostridium difficile BI1 | transketolase | ***-3.056*** | ***3.527*** |  |
| CDBI1_13050 | CDBI1_13050 | Peptoclostridium difficile BI1 | pyruvate-flavodoxin oxidoreductase | ***-3.069*** | ***2.510*** |  |
| CDBI1_16960 | CDBI1_16960 | Peptoclostridium difficile BI1 | F0F1 ATP synthase subunit beta | ***-3.176*** | ***2.746*** |  |
| CDBI1_15505 | CDBI1_15505 | Peptoclostridium difficile BI1 | glyceraldehyde-3-phosphate dehydrogenase 2 | ***-3.176*** | ***2.528*** |  |
| tig | CDBI1_16215 | Peptoclostridium difficile BI1 | trigger factor | ***-3.176*** | ***1.358*** |  |
| CdifQCD-_020200013241 | CdifQCD-_020200013241 | Clostridium difficile QCD-97b34 | pyruvate-flavodoxin oxidoreductase | ***-3.242*** | ***2.018*** |  |
| rplK | CDBI1_00350 | Peptoclostridium difficile BI1 | 50S ribosomal protein L11 | ***-3.269*** | ***1.470*** |  |
| CDBI1_00930 | CDBI1_00930 | Peptoclostridium difficile BI1 | redox-sensing transcriptional repressor Rex | ***-3.295*** | ***2.421*** |  |
| CDBI1_03375 | CDBI1_03375 | Peptoclostridium difficile BI1 | aminoacyl-histidine dipeptidase | ***-3.415*** | ***1.998*** |  |
| CDBI1_00265 | CDBI1_00265 | Peptoclostridium difficile BI1 | prolyl-tRNA synthetase | ***-3.442*** | ***2.961*** |  |
| thiH | CDBI1_10450 | Peptoclostridium difficile BI1 | thiamine biosynthesis protein ThiH | ***-3.468*** | ***1.650*** |  |
| greA | CDBI1_17400 | Peptoclostridium difficile BI1 | transcription elongation factor GreA | ***-3.614*** | ***2.091*** |  |
| CDBI1_03405 | CDBI1_03405 | Peptoclostridium difficile BI1 | adenosylcobamide-dependent radical SAM protein | ***-3.628*** | ***1.344*** |  |
| CDBI1_01505 | CDBI1_01505 | Peptoclostridium difficile BI1 | hypothetical protein CDBI1_01505 | ***-3.760*** | ***1.590*** |  |
| CDBI1_16090 | CDBI1_16090 | Peptoclostridium difficile BI1 | formate acetyltransferase | ***-3.960*** | ***1.373*** |  |
| CDBI1_13380 | CDBI1_13380 | Peptoclostridium difficile BI1 | adenine phosphoribosyltransferase | ***-4.053*** | ***1.476*** |  |
| rplF | CDBI1_00485 | Peptoclostridium difficile BI1 | 50S ribosomal protein L6 | ***-4.265*** | ***2.321*** |  |
| CDBI1_17105 | CDBI1_17105 | Peptoclostridium difficile BI1 | DNA-binding protein HU | ***-4.823*** | ***2.403*** |  |
| CDBI1_19448 | CDBI1_19448 | Peptoclostridium difficile BI1 | hypothetical protein CDBI1_19448 | ***-6.298*** | ***1.328*** |  |
| CDBI1_05205 | CDBI1_05205 | Peptoclostridium difficile BI1 | ribonuclease g | ***-6.405*** | ***1.400*** |  |
| CDBI1_00450 | CDBI1_00450 | Peptoclostridium difficile BI1 | 50S ribosomal protein L29 | ***-6.431*** | ***1.509*** |  |
| CDBI1_01190 | CDBI1_01190 | Peptoclostridium difficile BI1 | dtdp-4-dehydrorhamnose reductase | ***-6.498*** | ***1.484*** |  |

Red indicates high abundance; green indicates less abundance. Increasing fold changes are reported as brighter color.

Fold changes reported in Log2 values.

P-values reported as -log10 values; a P value higher than 2 denotes very high significance.


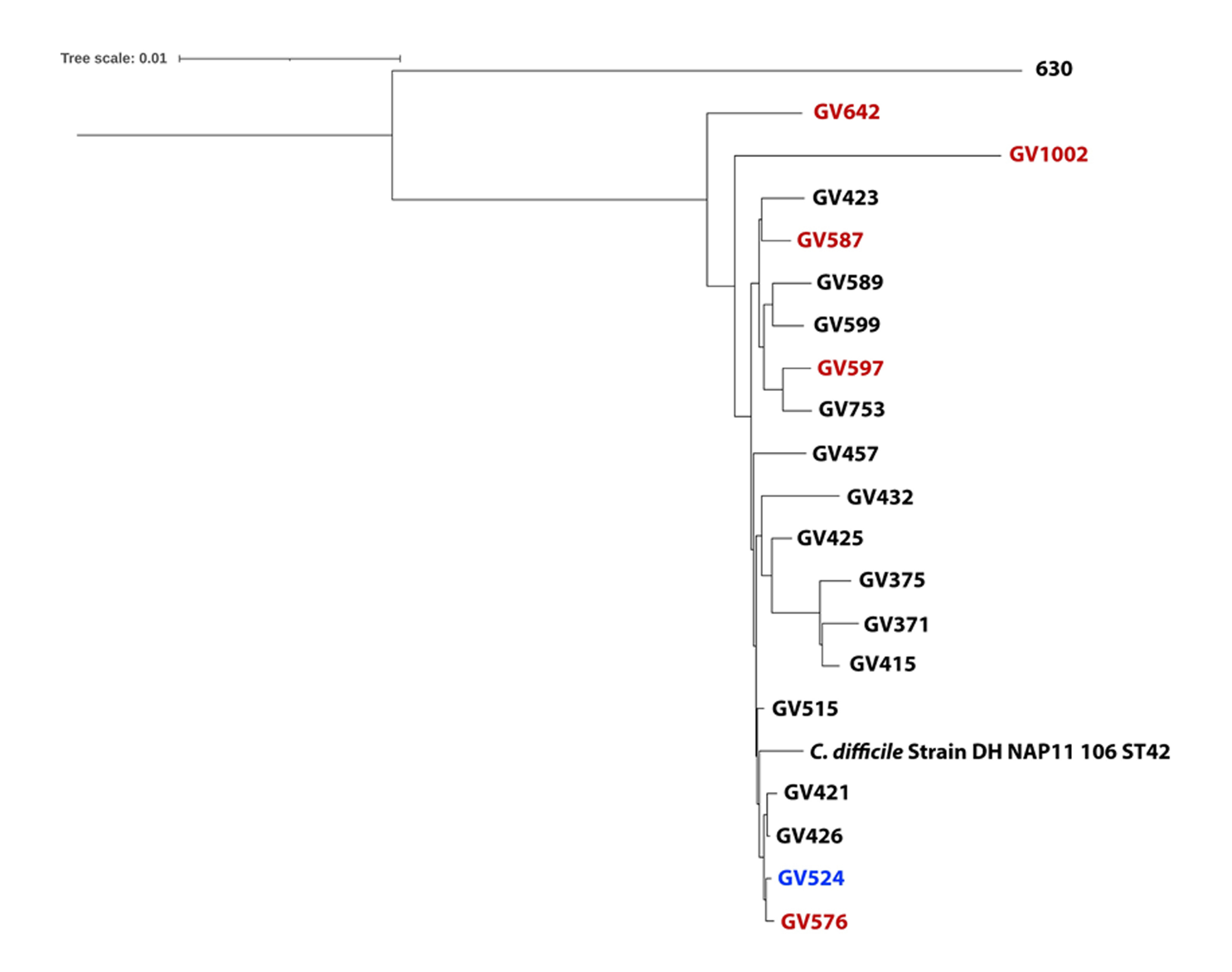


**Supplemental Figure S4: Composite Vector Tree of High- and Low-Toxin RT106 Strains**

Whole genome comparison was done between clinically derived, low-toxin producing (n = 5; red) and high toxin producing strains (n = 14; black). Strain 630 is a low-toxin comparator and GV524 is a Tox^-^/NAAT^-^ strain. Whole genome comparison of these strains show that low-toxin RT106 strains do not clade separately and are interspersed between high-toxin strains.


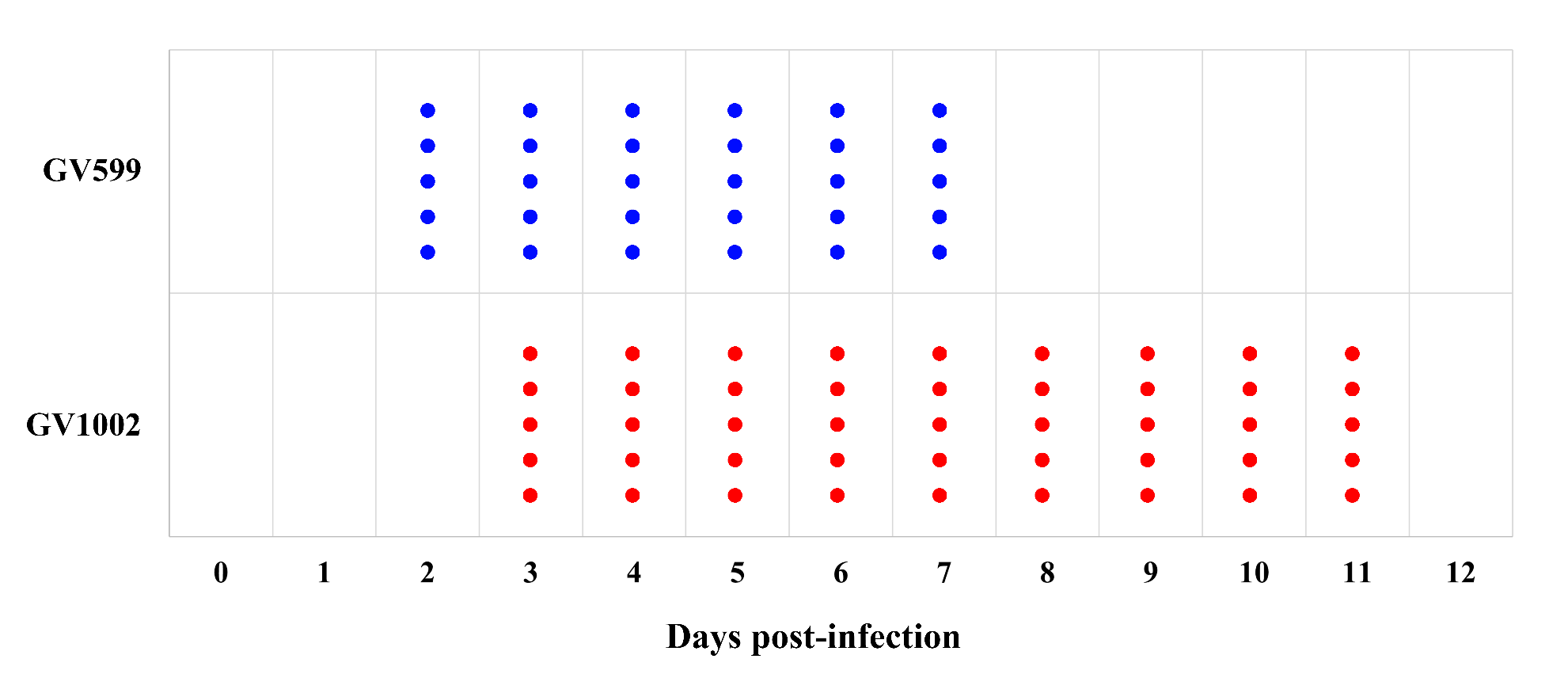


**Supplemental Figure S5: Mouse Infection with High- and Low-toxin RT106 Strains**

C57L/6 mice (n = 5 / group) were infected with a high-toxin RT106 strain, GV599 (blue), or a low-toxin RT106 strain, GV1002 (red). Each dot represents an animal that had detectable *C. difficile* in the stool. The high-toxin strain GV599 was detected on Day 2 post-infection and subsequently cleared in all mice by Day 8 post-infection resulting in a 6-day infectious course. The low-toxin strain was detected on Day 3 post-infection and was subsequently cleared by all mice by Day 12 post-infection resulting in a 9-day infectious course.


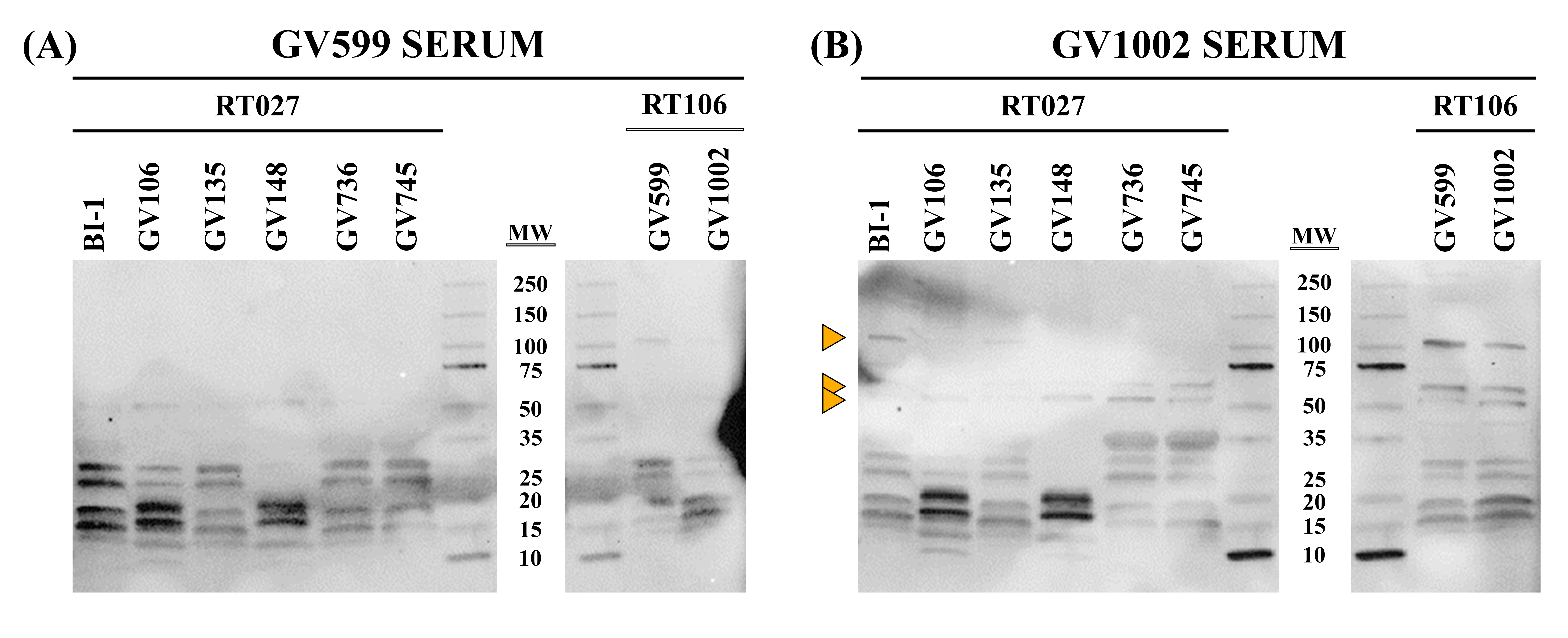


**Supplemental Figure S6: Membrane Fractions Probed with High- and Low-toxin RT106 Infected Mouse Serum**

Membrane extracts from high-toxin strains BI-1 (RT027) and GV599 (RT106), as well as the low-toxin strains GV106, GV135, GV148, GV736, GV745 (RT027s) and GV1002 (RT106) were probed with serum from mice infected with GV599 and GV1002. Similar to the results described for RT027-infected mouse serum (Figure 4), the low-toxin RT106 strain serum (GV1002) recognizes distinct bands that are not evident when probing with high-toxin strain serum (GV599; right-hand side blot in Panels A and B). Additionally, the GV1002-derived serum recognizes distinct protein bands in all strains irrespective of toxin status (orange arrows) suggestive of conserved presence or surface display of conserved moieties only in low-toxin strains.

**Supplemental Table S9: Discrepant RT106-specific Genes Compared to Discrepant RT027 Genes.**

| **Gene ID** | **AA Length** | **Function** | **Found In LT027 Pangenome?** | **Found in GV148?** | **Found in BI-1?** |
| --- | --- | --- | --- | --- | --- |
| fig\|1496.6344.peg.314 | 913 | PAS domain histidine kinase | N | N | N |
| fig\|1496.6344.peg.709 | 187 | Mobile element protein | N | N | N |
| fig\|1496.6344.peg.831 | 98 | Mobile element protein | N | N | N |
| fig\|1496.6344.peg.1058 | 308 | hypothetical protein | N | Y | Y |
| fig\|1496.6344.peg.1149 | 1129 | Transcription-repair coupling factor | N | Y | Y |
| fig\|1496.6344.peg.1255 | 123 | Functional domain: RNA-binding protein YhbY | Y | Y | N |
| fig\|1496.6344.peg.1360 | 39 | hypothetical protein | N | N | N |
| fig\|1496.6344.peg.1711 | 458 | Arginine utilization regulatory protein RocR | N | Y | Y |
| fig\|1496.6344.peg.1854 | 44 | LytR family transcriptional regulator domain | N | N | N |
| fig\|1496.6344.peg.1937 | 200 | hypothetical protein | N | Y | Y |
| fig\|1496.6344.peg.2047 | 898 | peptidoglycan transglycosylase (EC 2.4.1.129) | N | Y | N |
| fig\|1496.6344.peg.2128 | 637 | Phosphonoacetaldehyde hydrolase (EC 3.11.1.1) / 2-aminoethylphosphonate:pyruvate aminotransferase (EC 2.6.1.37) | N | Y | Y |
| fig\|1496.6344.peg.2343 | 50 | hypothetical protein | N | N | N |
| fig\|1496.6344.peg.2544 | 289 | FIG00512787: hypothetical protein | N | Y | Y |
| fig\|1496.6344.peg.2729 | 445 | Pyridine nucleotide-disulfide oxidoreductase; NADH dehydrogenase (EC 1.6.99.3) | N | Y | Y |
| fig\|1496.6344.peg.2831 | 44 | hypothetical protein | N | N | N |
| fig\|1496.6344.peg.3114 | 699 | CocE/NonD family hydrolase | N | Y | Y |
| fig\|1496.6344.peg.3564 | 425 | Two-component sensor histidine kinase | N | Y | Y |

Discrepant RT106 strains (N=5); non-discrepant RT106 strains (N=4)
